# Supplementary material for: Laminar differences in decision-related neural activity in dorsal premotor cortex
Source: Nat Commun. 2017 Sep 20;8:614. doi: 10.1038/s41467-017-00715-0 (PMC5607001; doi:10.1038/s41467-017-00715-0)
Supplement: Supplementary file 1 — Supplementary Information [file 41467_2017_715_MOESM1_ESM.pdf]

File Name: Supplementary Information
Descriptions: Supplementary Figures, Supplementary Tables, Supplementary Notes and Supplementary References

File Name: Peer Review File

Supplementary Figures, Notes, Discussion

Supp. Fig. 1 - A drift diffusion model of the behavior

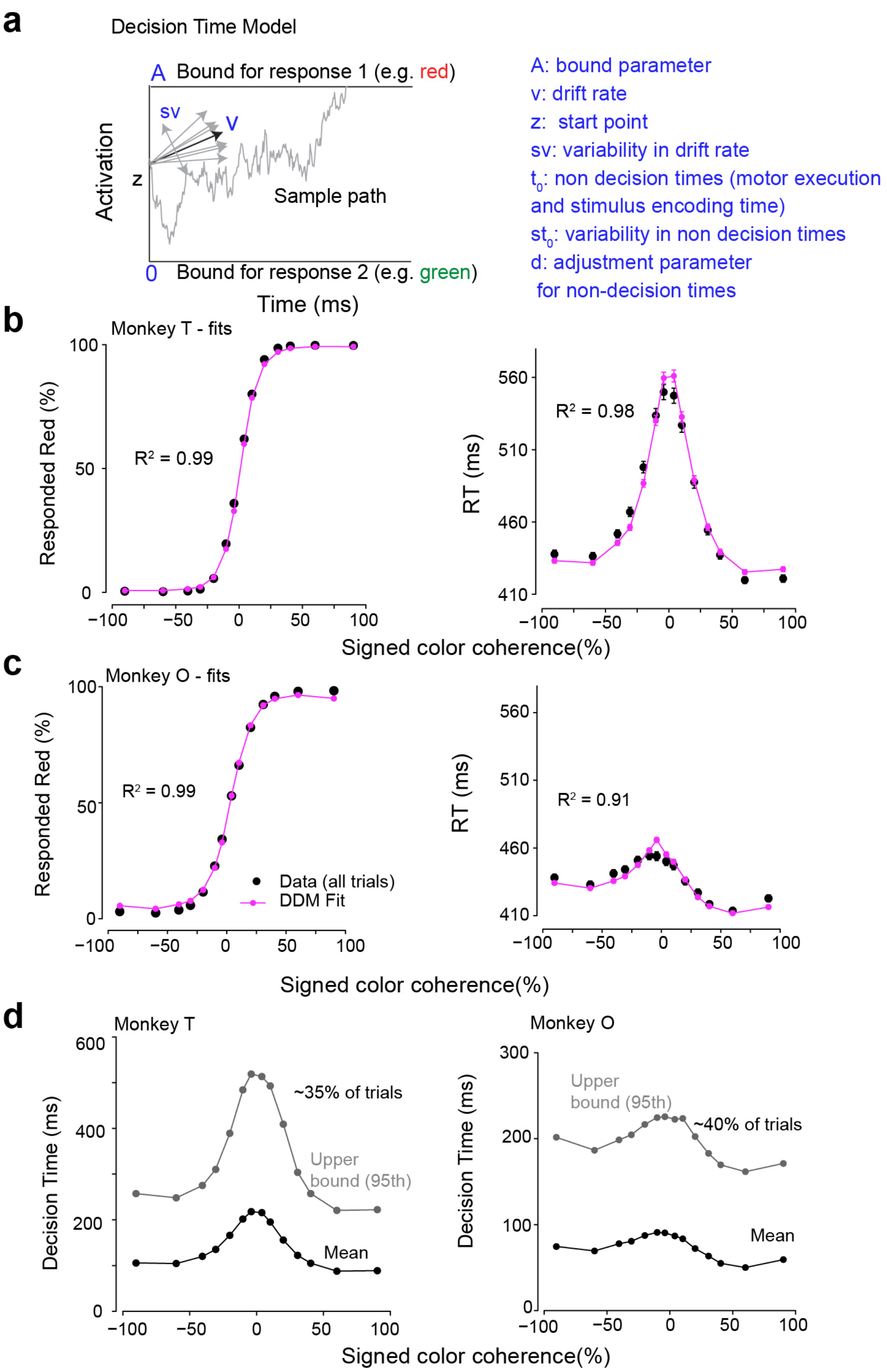

Supplementary Figure 1: A drift-diffusion model can model the discrimination behavior of the monkeys.

a: A schematic illustration of a sample path in the drift-diffusion model of a perceptual decision. On every trial, the evidence for one of the choices (say red) over the other choice (i.e., green) is accumulated over time. Different mean rates of accumulation (v) parameterize the evidence for each of the stimulus strengths. When the process reaches one of the bounds (0 or A), the decision is made. The DDM only models the decision process. The complete reaction time is described as the decision time estimated from the DDM plus an additive non-decision time (t_0) that is thought to reflect processes such as stimulus encoding and motor preparation. Other factors such as slightly different non-decision times for different responses (d), variability in drift rates (s_v), and variability in non-decision times (st_0) are included to fit RT variability.

b: Fits of the DDM to the percent of red choices (left panel) and RTs (right panel) as a function of signed color coherence for monkey T. In the left panel, the black filled dots depict proportion responded red measured from the experimental data, the magenta dots depict the fits from the DDM. The predicted points in magenta are obtained by simulating 10000 trials from the DDM for each data point. Magenta line segments are drawn in between model predicted points to guide the eye. Psychometric curves and RT curves are obtained by pooling over 75 sessions for monkey T (128989 trials). In the right panel, the magenta points show the predicted mean RTs from the DDM simulation (10000 simulated trials with the estimated parameters) along with the measured RT in black filled dots. Magenta line segments are again drawn in between the predicted points to guide the eye. Error bars in the right panel denote 3*SEM estimated over trials (measured RTs) or simulations (for the DDM). X-axes in both panels depict signed color coherence. Y-axes depict either percent responded red (left panel) or RT (in ms) in the right panel. The proportion of variance explained is computed over 14 points for each curve.

c: Fits of the DDM to behavior from monkey O. The fits to the RT data for monkey O are again excellent albeit not as good those for monkey T. Conventions as in b. Data are fits from pooled trials over 66 sessions for monkey O (108344 trials). Proportion of variance is computed over 14 points.

d: Mean and upper bound (95th percentile) of the decision time estimated by subtracting the estimated non-decision times from the mean and 95th percentile of the RT distributions for each level of signed color coherence in the stimulus. We estimated the range of decision times by fitting the model to pooled data and subtracting non-decision time from the mean and 95th percentile of the pooled RT distribution. An increase in color coherence results in shorter decision times. X-axes depict signed color coherence; Y-axes depicts decision time in ms.

Supp. Fig. 2 - Comparing the fits of the DDM and the UGM to the behavior of the monkeys

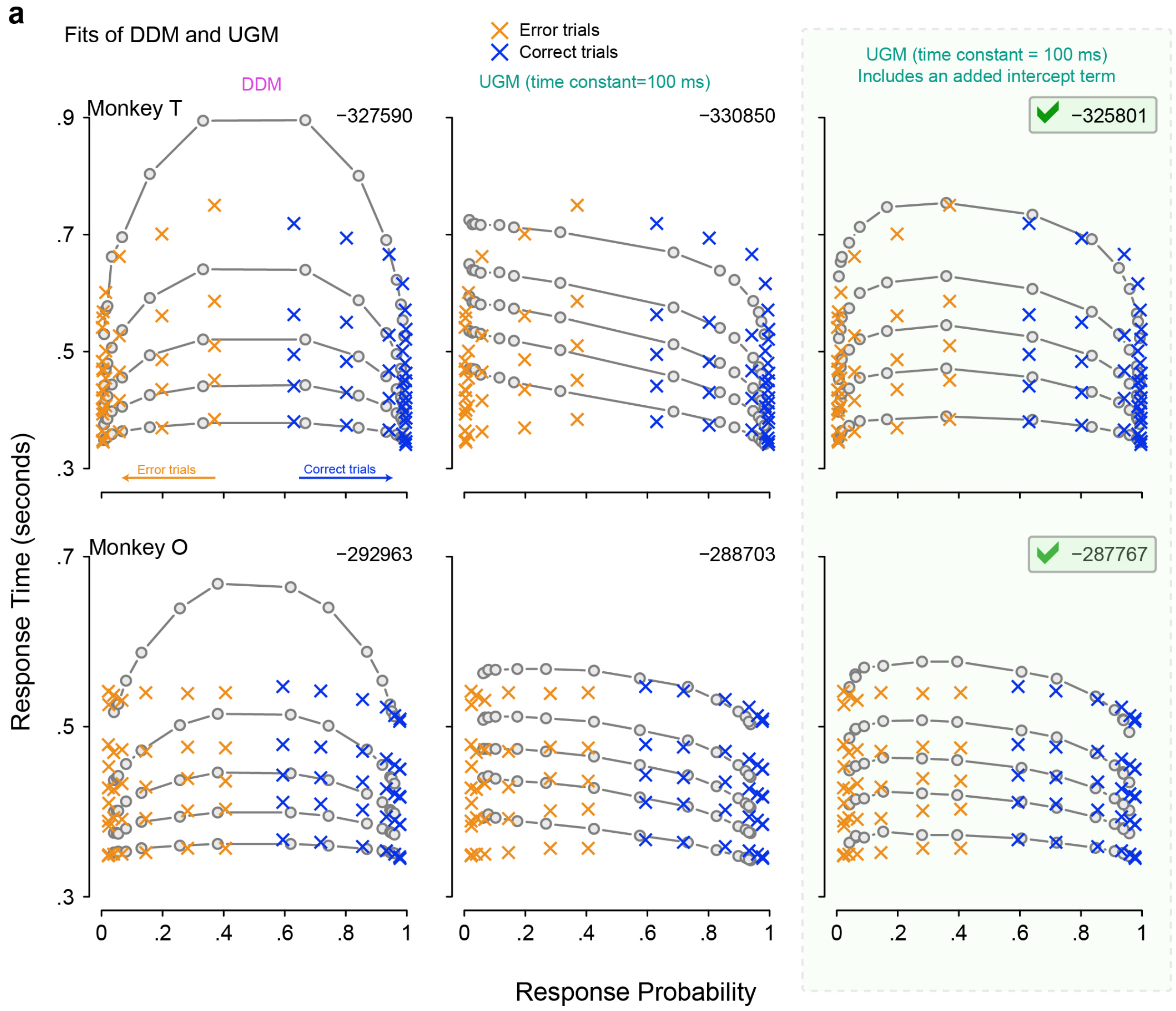

Supplementary Figure 2: Modeling RT and accuracy of the monkeys using both a DDM and UGM

a: Fits of either the DDM (left panel), the UGM (middle panel), or the UGM with an intercept (right panel) to the behavior of monkey T and monkey O. RT and accuracy are depicted using quantile probability plots [1, 2]. The crosses show the real data with correct trials shown in blue and the incorrect trials shown in orange. The gray dots and lines show the predictions. The gray lines are again provided to guide the eye. A goodness of fit statistic termed quantile maximum products estimate (QMPE) is also provided [3]. Values closer to zero are better for this statistic. Note that the DDM can replicate the shape of the plot but overinflates the RTs for the highest quantiles. The UGM without an intercept term misses on shape but does not predict very large RTs. Finally, the UGM with an intercept term (an additional parameter over the models shown in the center and left panels) provides the best description of the behavior of the monkeys as provided by the QMPE statistic. The green ticks and shading denote the model that provided the better fit.

Supp. Fig. 3 - Visuomotor index for each monkey separately

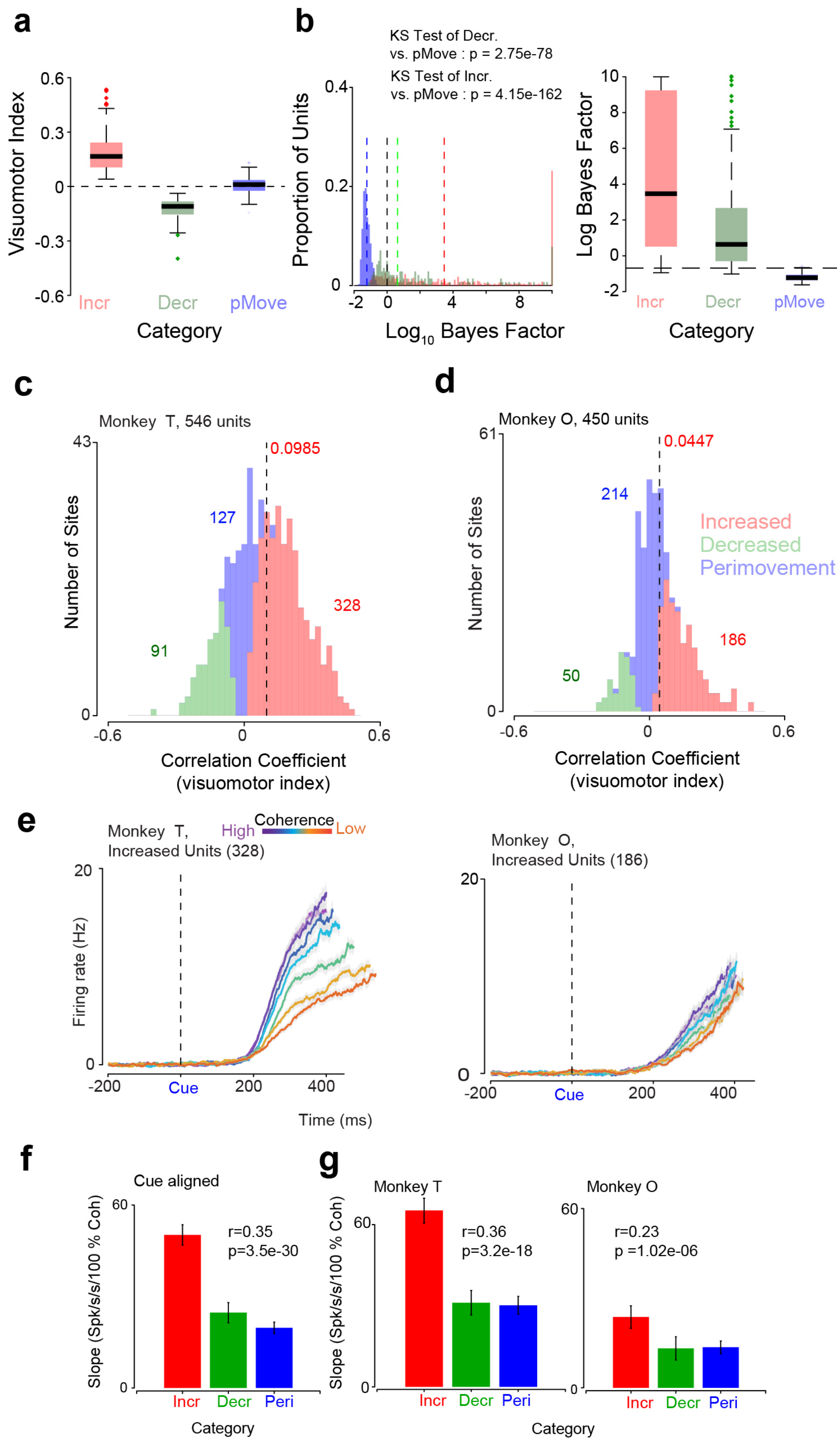

53

Supplementary Figure 3 – Visuomotor indices and dependence on coherence shown separately for each monkey

a: Box plot showing the distribution of visuomotor index for the three broad neural categories of interest. X-axes depict category. Y-axes depict visuomotor index. Whiskers show the 5th and 95th percentile confidence intervals.

b: Left panel, Histogram of log10(BayesFactor) for the visuomotor index. Log Bayes Factors larger than 10 were included in the final bin of the histogram. A Kolmogorov Smirnov test which examined the overlap between the distributions of Bayes Factors for the increased vs. perimovement and increased vs. decreased units was robustly significant. The right panel shows a box plot of the Bayes Factor for the three different conditions. Very little overlap exists between the Bayes factors for these populations suggesting that our method of separation was reliable and very rarely classified perimovement units as increased or decreased units.

c,d: Histogram of the visuomotor index demonstrating broad unit categories in PMd shown separately for monkey T (**a**) and monkey O (**b**). Figure conventions as in Fig. 3a.

e: Average population level choice selective signal (|left – right|) in PMd for the population of increased units as a function of the color coherence aligned to checkerboard cue onset. All trials are included and sorted by the choice of the monkey. Figure conventions as in Fig. 3a. Monkey T is shown in the left panel and Monkey O in the right panel.

f, g: Dependence of the rate of rise of the choice selective signal in the 150–350 ms epoch after checkerboard cue onset on color coherence for the three broad unit classes (rate of rise of the curves shown in Fig. 3c or in Supp Fig. 3e). Increased units are modulated the strongest by the color coherence of the checkerboard cue. X-axes depict different categories. Y-axes depict the slope of the curve in Fig. 3c or in Supp Fig. 3e and is measured in Spks/s/s/100% coherence. The correlation coefficient between the rate of rise and the visuomotor index is shown in the figures. Panel f shows it for the pooled data, panel g shows it for each monkey separately.

Supp. Fig .4 - Additional statistics for Figure 3.

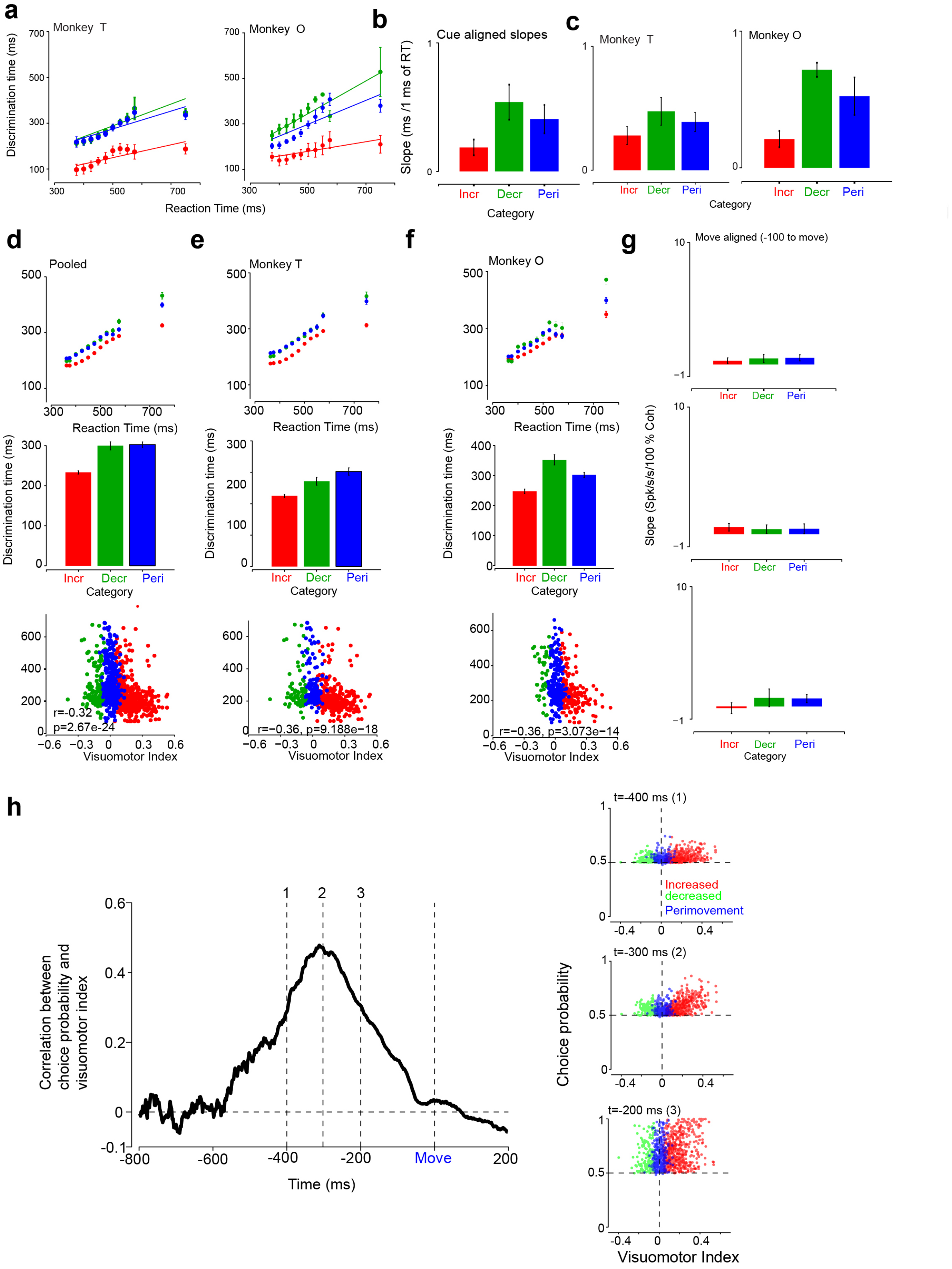

Supplementary Figure 4: Summary statistics for the population of units and per monkey

a: Population level discrimination time as a function of RT for each monkey separately for each of the broad unit categories. This figure complements Fig. 3e and shows that activity related to choice emerges at the population level earlier in the increased compared to the decreased and perimovement units.

b: Slopes of the discrimination time vs. RT lines shown in Fig. 3e when aligned to checkerboard cue onset. For increased units, the slope is smaller suggesting that regardless of eventual RT, choice related signals begin to appear in PMd approximately 150 ms after checkerboard cue onset. In contrast, for the perimovement and decreased units, information about choice is only available close to the movement onset and changes with RT. X-axes depict unit category. Y-axes depict slope in ms/1 ms of RT.

c: Same as **b** but with slopes computed for each monkey separately for the plots shown in Supp. Fig. **4a**.

d: Top panel shows the average discrimination time as a function of RT bin estimated on a *neuron by neuron* basis and then averaged for each of the three broad unit categories. The middle panel shows the average discrimination time for all RTs ranging from 300 to 1000 ms for each broad unit category. The bottom panel shows a scatter plot of the visuomotor index vs. the discrimination time colored by the category of the unit. There is a significant negative correlation between the index and the time of discrimination.

e, f : Same as d for monkey T (e) and monkey O (f). Figure conventions as in d.

g: By 100 ms before movement onset, the slope of the choice selectivity signal only modestly depends on color coherence. Slopes are estimated on the lines shown in Fig. 3h and compared to a control slope estimated by shuffling FR across the color coherences. X-axes depict unit category. Y-axes depict slope of curves in Fig. 3h and is measured in Spks/s/s/100% coherence. The bottom panels show this separately for monkeys T and O.

h: Choice selectivity is distributed in the population. The left panel shows the correlation between choice probability estimated at each time point for the population of 996 units and the visuomotor index. X-axes depict time in ms. Y-axes depict the correlation coefficient. The right panel shows a scatter plot between choice probability and visuomotor index for three different time points (-400, -300, -200 ms before movement onset). In all three cases, it appears to be more consistent with a continuum than discrete clusters.

Supp. Fig. 5 - Another method for estimating a visuomotor continuum

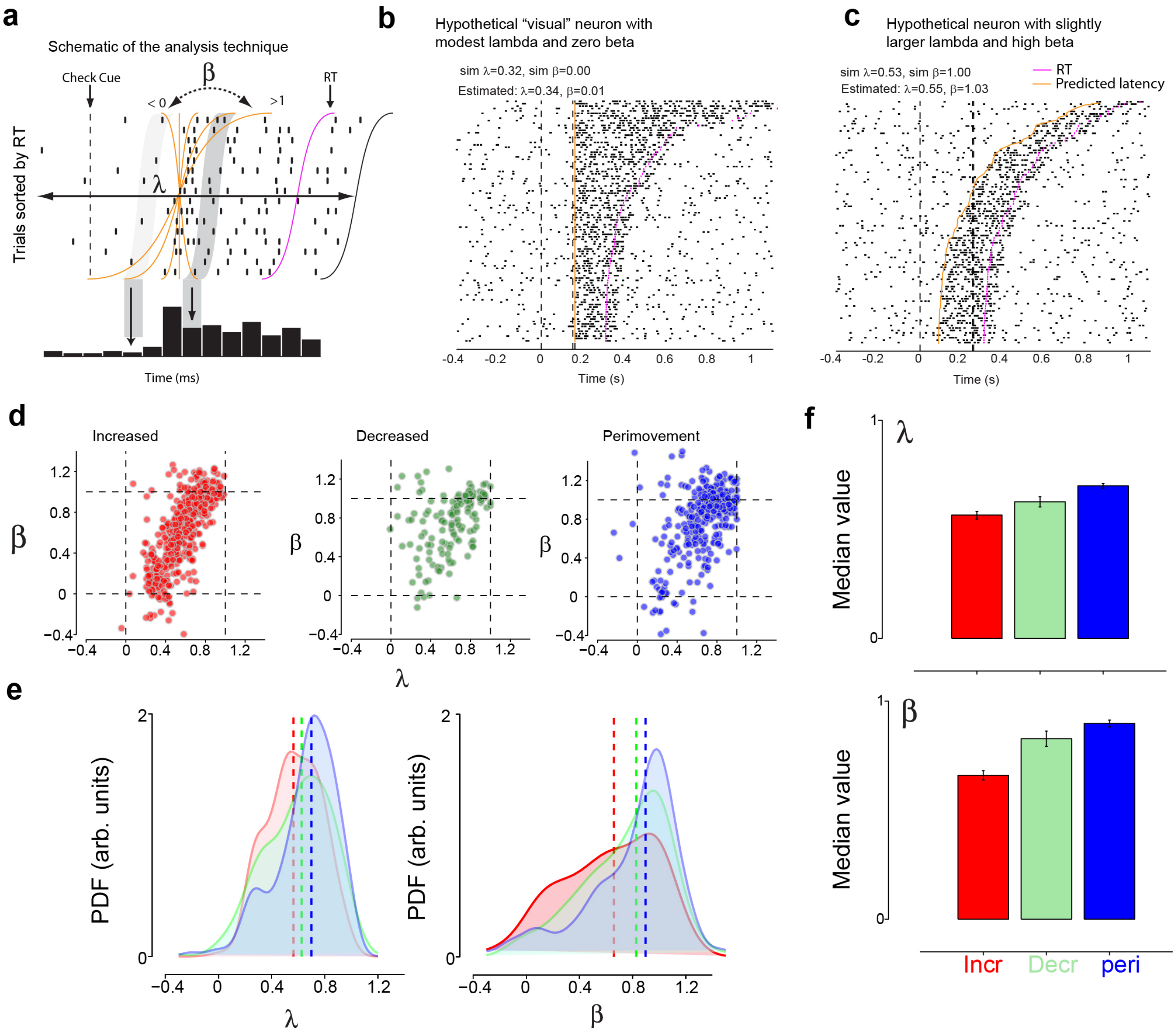

Supplementary Figure 5 – The index is consistent with other indices proposed for characterizing a sensorimotor processing chain

a: A schematic of the analysis technique proposed in [4] to characterize this visuomotor continuum. The objective of this technique is to estimate the position and slope of a rate change line parameterized by two terms λ and β so that the mean FRs on each side of the line (over all trials) is most different. λ is a normalized latency measure (defined as $\frac{\mu_{NL}}{\mu_{RT}}$). This parameter is the location of the center of the rate-change line. β is a parameter that deforms the line between complete vertical to entirely match the shape of the RT distribution and is a constant of proportionality that shifts each trial in time according to the RT on that trial so that the spikes from all trials are brought into optimal alignment. We used the least squares error technique described in [4] to find the rate change line that yields the smallest error for the rates over the surfaces on either side of the line. This figure is redrawn from Fig. 2 of Ref. [4].

b: A hypothetical neuron simulated with a modest λ (0.32) and β=0**.** This hypothetical neuron is consistent with a classical "visual" neuron that shows minimal covariation with RT and only responds to the onset of the visual stimulus. The estimated values of λ and β are very close to the simulated values. Magenta tick marks depict RT. The red line depicts the estimated line for each trial. The dashed line shows the simulated latency for the hypothetical neuron.

c: A hypothetical neuron simulated with a slightly higher λ **(0.53)** and almost perfect covariation with RT (β=1). Figure conventions as in **b**.

d: λ and β estimated for **e**very increased (left), decreased (middle), and perimovement (right) unit in our database. The increased units appear to span the entire continuum whereas the perimovement units are generally closer to β=1. The decreased units are somewhere in between. The plotted values of **λ and β** are averaged over both left and right reach choices**.**

e: Probability densities of λ and β for the increased, decreased and perimovement units. Lines show the median values for each of the different units. The y-axes depict probability density in arbitrary units so that the integral of the probability density function is equal to 1.

f: Median λ and β for each of the broad unit categories. The increased units have the lowest medians of λ and β and these are significantly different from the λ and β values for the decreased and perimovement units. X-axes depict broad unit category. Y-axes show the median value.

Supp. Fig. 6 - Visuomotor Index is consistent with more sophisticated metric for estimating correlation between RT and neural responses

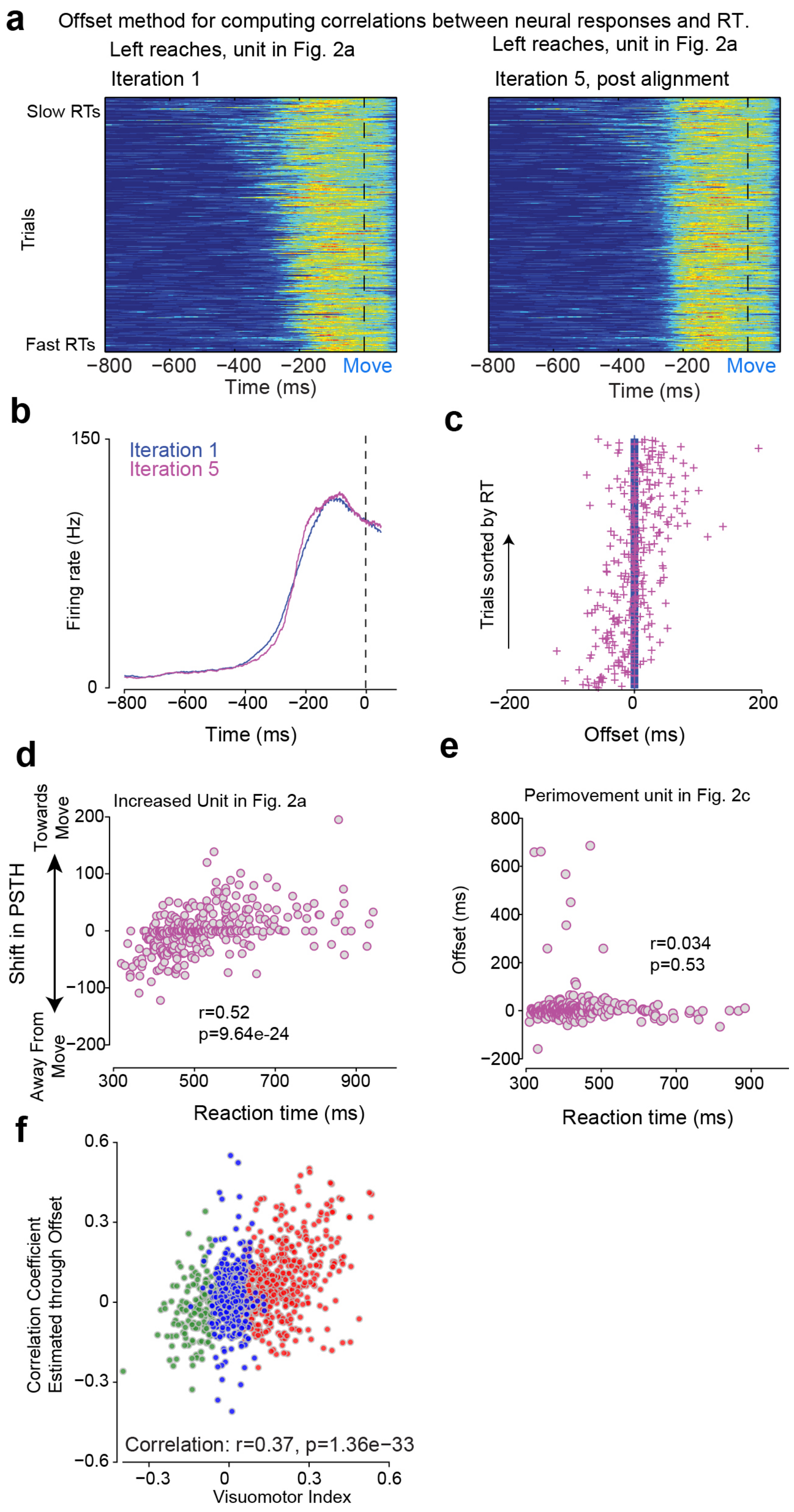

Supplementary Figure 6 – The index is tightly correlated with a more sophisticated measure of correlation between neural responses and RT

a: Left panel. PMTH for left reaches for the neuron shown in Fig. 2a aligned to movement onset for all trials. X-axes depict time in ms. Y-axes the trials sorted from fast to slow RTs. This is the input to the iterative algorithm developed in ref. [5] for relating complex neural responses to RT. Right, same trials now shifted according to the offset method. Note the better alignment of the peak of the FRs by adjusting each trial according to an appropriate offset.

b: Mean PMTH aligned to movement onset in the first iteration of the algorithm and the 5th iteration of the algorithm.

c: The offset for each trial sorted by the RT. Responses for faster RTs need to be shifted away from movement onset (by adding a negative offset here). The slower RT responses are shifted towards movement onset (by adding a positive offset).

d: Plot of RT vs. temporal offset. There is a significant positive correlation between the offset and RT for the increased unit shown in Fig. 2a.

e: Same plot as in Fig. 2d but for the perimovement unit shown in Fig. 2c. Conventions as in Fig. 2d. The points show RT and offset for the right reach trials)

f: Plot of a sign corrected correlation coefficient (averaged over both reach directions) estimated from the technique and the visuomotor index. Red circles depict putative increased neurons; green predicts decreased neurons, and blue depicts perimovement neurons. The sign correction was performed because the offset method does not take into account whether the neuron increases or decreases its FR. Note the strong correlation between the simple visuomotor index and this more sophisticated correlation between RT and neural responses.

Supp. Fig. 7 - Slope analyses as in Song and McPeek, 2010

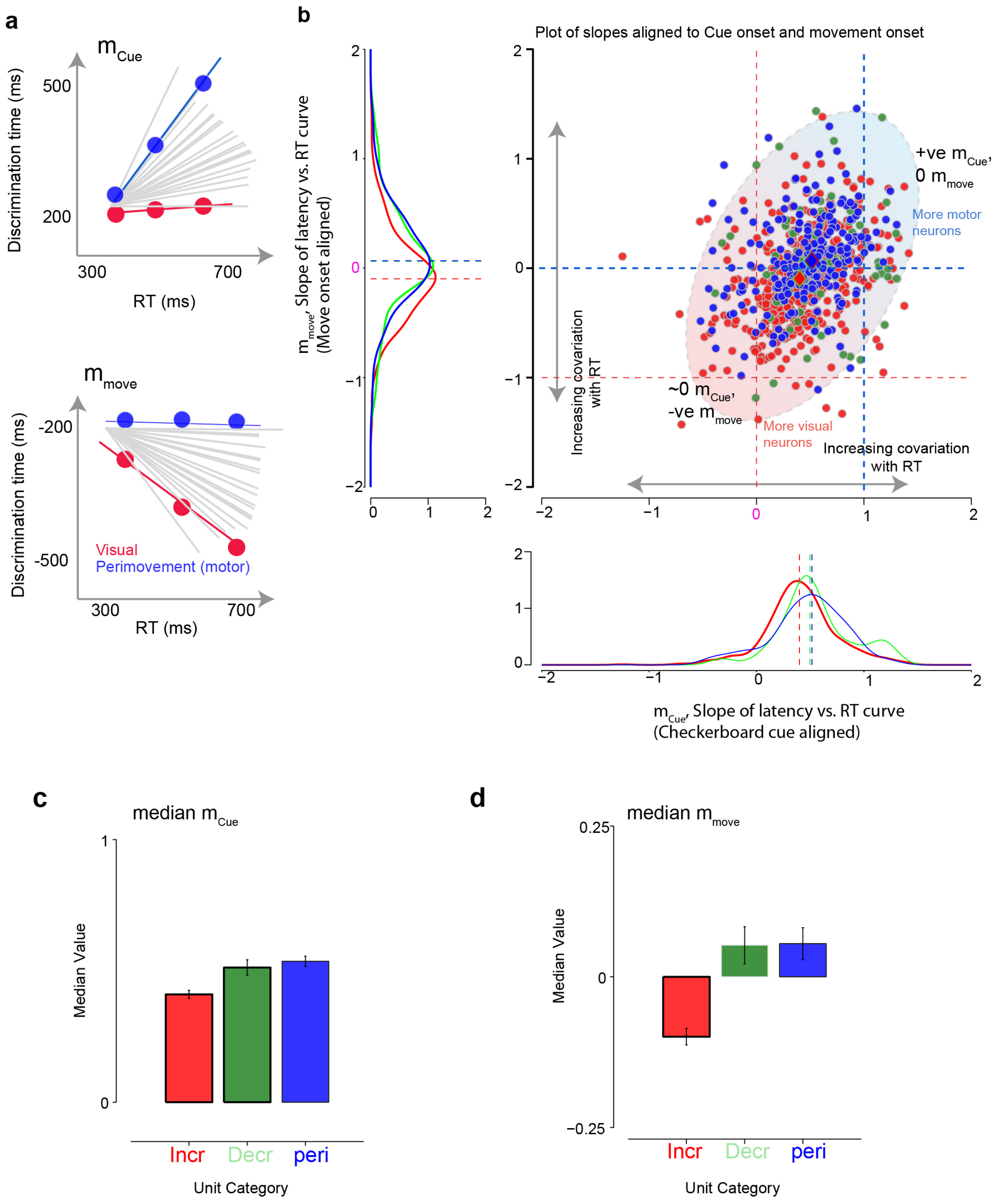

181

182

Supplementary Figure 7 – Analysis of the dependence of discrimination time on RT supports a visuomotor continuum.

a: A schematic illustrating the values of m_{cue} and m_{move} for hypothetical purely visual and purely motor neurons. The parameter of interest is the slope of the curve between discrimination time and RT when aligned to either checkerboard cue onset or movement onset. The perfect visual neuron would have a near zero slope (m_{cue}) when aligned to checkerboard cue onset and a slope almost equal to -1 when aligned to movement onset (m_{move}).

b:Scatter plot of m_{cue} and m_{move} for the population of units in PMd. The ellipse is drawn to guide the axis along which variation is expected. There seems to be a weak tendency for the neurons identified by the visuomotor index to be increased as having lower values of m_{cue} and more negative m_{move}. The more "motor" neurons appear to have slightly higher values of $m_{cue\ and}$ m_{move} slopes closer to 0.

c: Median m_{cue} for the population of neurons sorted by broad unit category. X-axes show broad unit category. Y-axis the median value for the slope aligned to checkerboard cue onset.

d: Median m_{move} for the population of neurons sorted by broad unit category. X-axes show broad unit category. Y-axis the median value for the slope of the discrimination time function aligned to movement onset.

Supp. Fig. 08 - Principal Components Analysis is consistent with a structure population

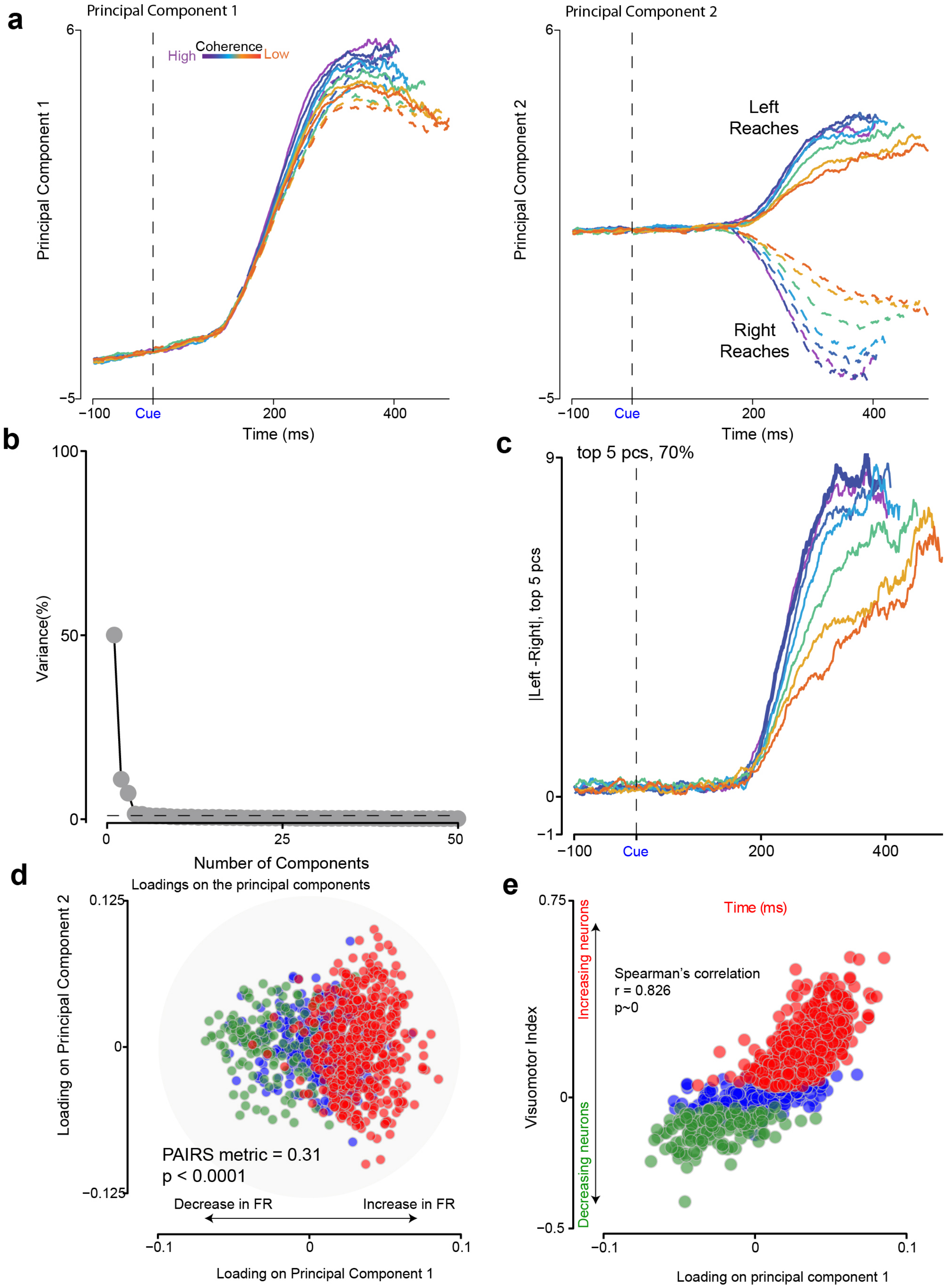

Supplementary Figure 8: Principal component analysis suggests that increased PMd neurons show the strongest projection on an axis strongly selective for choice and also support the broad separation into increased, decreased, and perimovement neural populations

a: 1st and 2nd principal component estimated from PCA on the population of FRs (50 ms boxcar) aligned to checkerboard onset and organized by coherence. X-axes depict time in ms. Y-axes depict the magnitude of the PC in normalized units. Colors denote different levels of coherence. Solid lines depict left reaches, dashed lines depict right reaches. PC1 involves a general change in FR with modest separation by choice and coherence. PC2 involves a lawful organization by choice and stimulus coherence.

b: Variance explained by each principal component. The top 4 PCs explain the majority of the variance. The dashed line depicts 1% of variance explained and is provided as a reference.

c: Difference in left vs. right for the top 5 PCs that explain ~ 71% of the FR variance in PMd during a decision-making task.

d: Loading on the first and second PCs obtained from the principal component analysis. Individual markers denote different units colored according to their broad unit category identified by the visuomotor index. Both the PAIRS statistic and a chi-square test rejected the hypothesis that loadings are uniformly distributed.

e: Plot of the visuomotor index vs. projection on PC1 for the population of PMd units that we examined. Again different markers are colored by their different unit categories. Note the robust spearman's correlation between the two metrics. PC1, as seen in panel c, explains ~50% of the variance and thus by proxy, the simple visuomotor index captures ~50% of the variance.

Supp. Fig. 09 - K Means Clustering

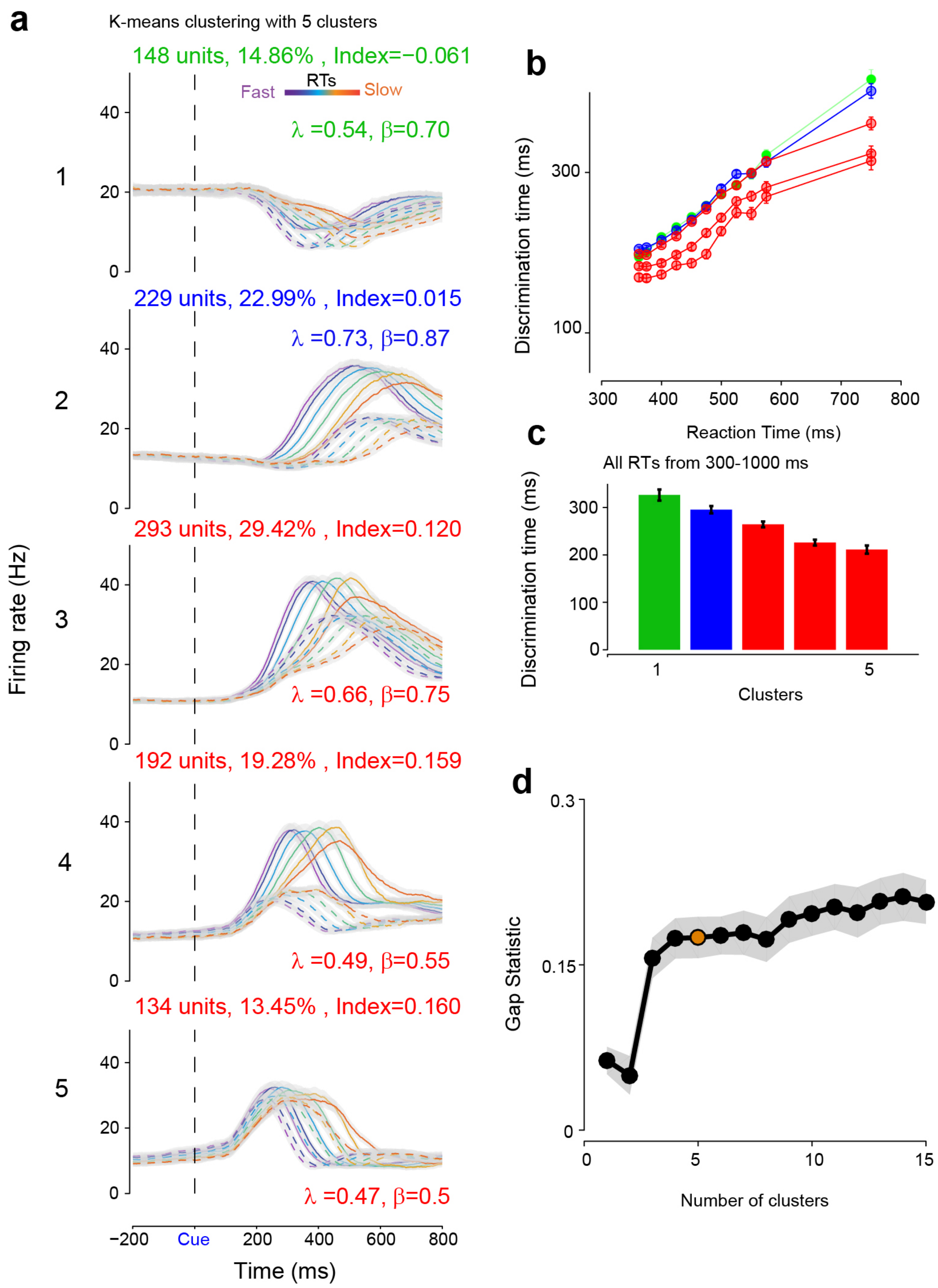

Supplementary Figure 9: A clustering analysis reveals that a modest number of clusters provide an excellent account of many of the FR patterns we observed

a: PSTHs from 5 different clusters identified by K-means for the preferred (PREF, solid lines) and nonpreferred (NONPREF, dashed line) directions as a function of time aligned to checkerboard cue onset. Inputs to the clustering algorithm were PSTHs aligned to checkerboard cue onset for PREF. and NONPREF directions averaged over all RTs. We included 100 ms before checkerboard cue onset and 600 ms after checkerboard cue onset sorted by tuning direction for the clustering analysis. We used a correlation measure as a distance metric and thus did not need any normalization. X-axes depict time in ms. Y-axes depict FR in Hz. Different colored lines denote different RTs as in Fig. 2a. Dashed black line denotes the onset of the checkerboard cue. The number of units in the cluster along with the average visuomotor index for these units is shown in the top of each panel. In each figure, we also include the λ and β indices that were developed in ref. [4]

b: Discrimination time as a function of RT bins for each of the clusters identified in a. Note that the units which increase their FRs after checkerboard cue onset discriminate the choice earlier than the perimovement and decreased neurons.

c: Same as a but for all RTs from 300-1000 ms. Note that cluster 5 that is the furthest into the increased part of the continuum shows the earliest covariation with the choice.

d: Plot of the gap statistic as a function of the number of clusters. We chose 5 as the number of clusters to show because it was the first cluster after the “elbow” in the plot. The chosen value of K=5 clusters was within within 2*SE of the max in the gap statistic as our estimate of the number of clusters that could best explain this dataset. A higher number of clusters provided similar results to what is observed in this figure.

Supp. Fig. 10 - Regression to exclude contributions of nuisance variables

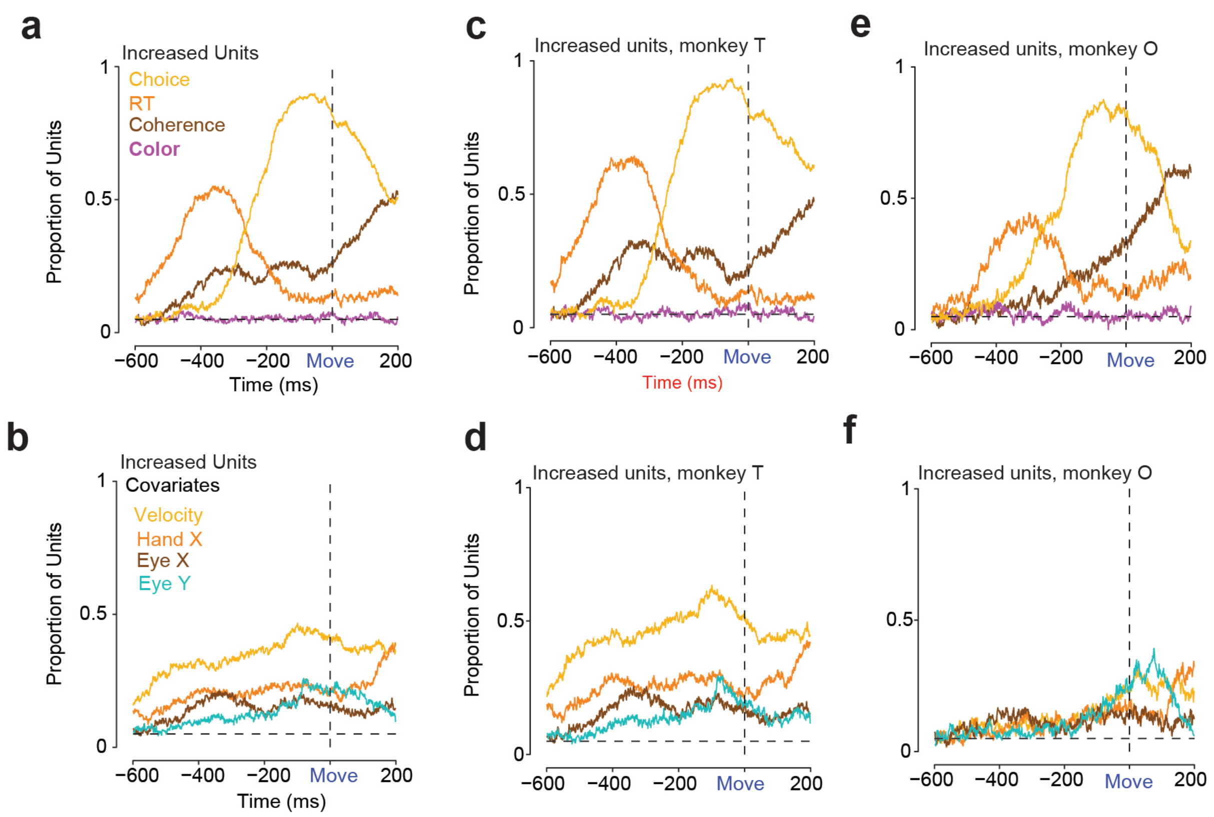

Supplementary Figure 10: Color of the target chosen is a poor predictor of FR in PMd, and instantaneous eye-position and hand position cannot entirely explain patterns of FRs.

a: The proportion of increased units with significant beta values for choice, RT, color coherence and color of target chosen when aligned to movement onset. This regression was performed while including the factors shown in b. X-axes depict time in ms aligned to movement onset. Y-axes depict the proportion of increased units for each predictor of interest. The FR of a unit at a time point was considered to be explained by a predictor if the 95% confidence intervals did not overlap with zero.

b: Same as a, except for hand position, reach speed, horizontal and vertical eye position. The proportion of units with significant beta values for choice, RT, and color coherence were computed from a regression where these nuisance covariates were included as predictors. X-axes depict time in ms. Y-axes depict the proportion of units with significant beta values for the covariates of interest.

c, e: Same as a for increased units from each monkey separately.

d, f: Same as b for increased units from each monkey separately.

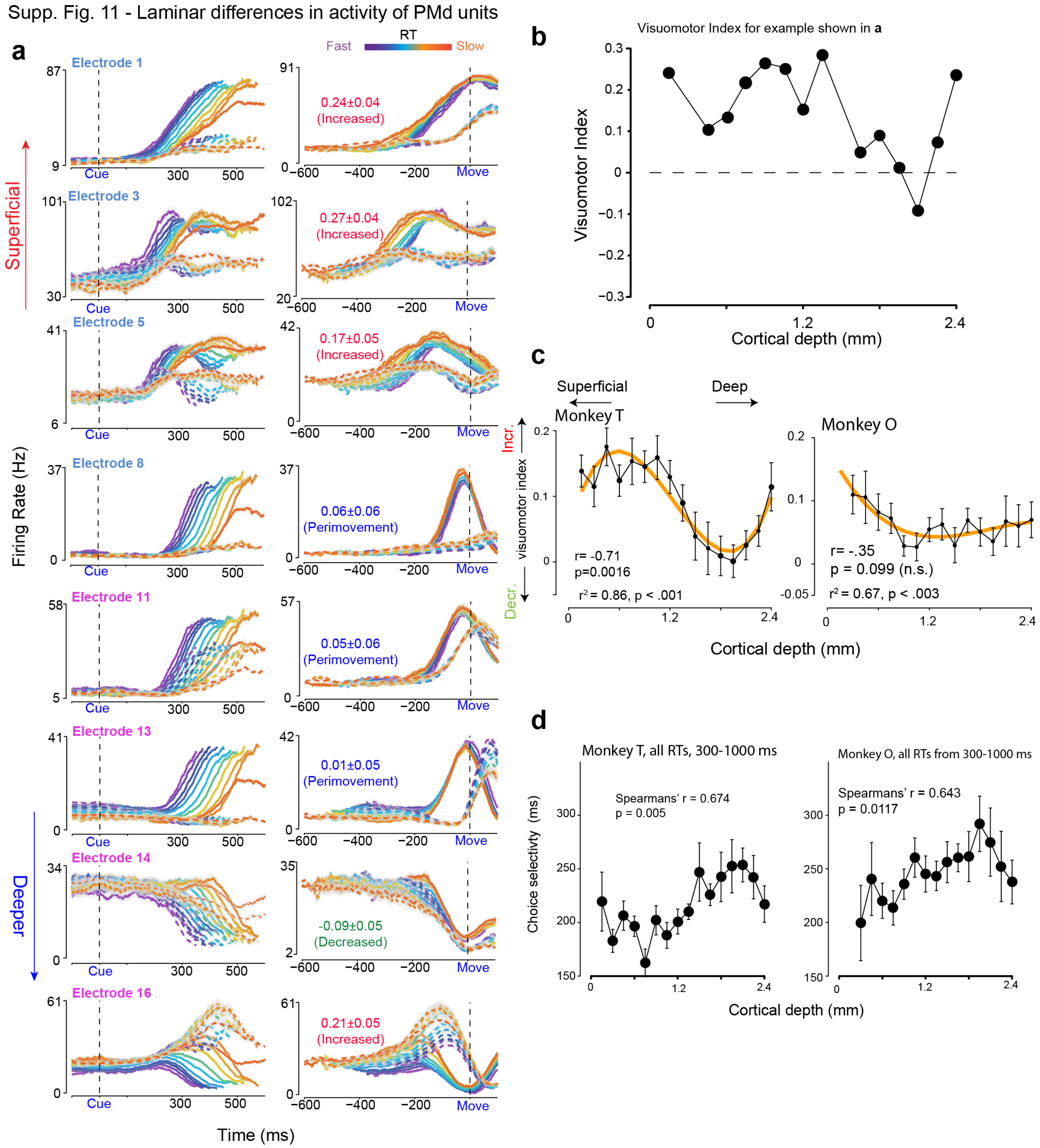

Supplementary Figure 11: An additional example suggesting a laminar organization in PMd during decisions

a: PSTHs from 8 different units recorded in the same session sorted as a function of RT and choice and ordered from superficial to deep electrodes. This is a different session from the one shown in Fig. 4. Figure conventions as in Fig. 4a. This single session example and the other single session example in Fig. 4a support the hypothesis that superficial electrodes have a greater tendency to

record from increased units (positive indices) whereas the deeper electrodes record more from perimovement and decreased units (negative and insignificant indices). For this session, PSTHs and PMTHs reflect FR averaged over more than 100 trials per condition.

b: Average visuomotor index as a function of cortical depth for the session whose examples are shown in Supp. Fig. 11a. Figure conventions as in Fig. 4b.

c: Average visuomotor index for monkey T and Monkey O as a function of cortical depth along with the best fit cubic line that describes the dependence of the visuomotor index on cortical depth. The coefficients along with the confidence intervals are as follows (Monkey T: a=134.7 (82.4, 187), **b = -44.2 (-62, -26)**, c = 3.0 (1.38, 4.79), d = 0.10, (0.06, 0.14); Monkey O: a=-26.9 (-71.9, 18.2), b = 14.17 (-2.25, 30.6), **c = -2.19 (-3.91, -0.46)**, d = 0.14, (.098, .19)). We used this higher order fit because of the nonmonotonicity in the data that involve both increases and decreases. P-values for the r^2 reported here are based on shuffling the index as a function of cortical depth and calculating the surrogate r^2 values and identifying the number of shuffled r^2 that exceeded the measured r^2.

d: The average discrimination time increases with cortical depth. Figure conventions as in Fig. 4d.

Supp. Fig. 12 - Classifier Accuracy Per Monkey and Posterior Recordings

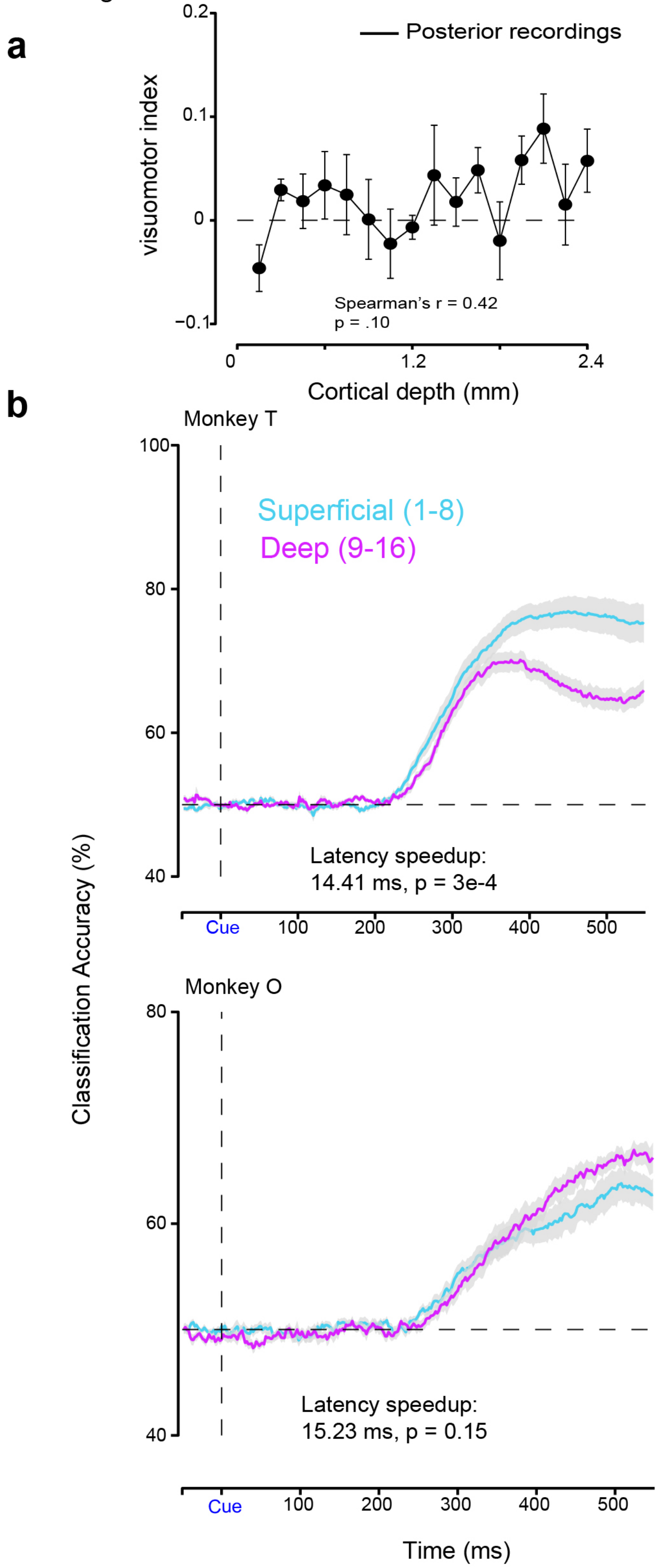

Supplementary Figure 12 – Caudal recordings and classification accuracy for PMd

a: Average visuomotor index as a function of cortical depth over 12 sessions recorded in a caudal location in monkey T's recording chamber (putatively M1). There is no systematic decrease as a function of cortical depth. X-axes depicts cortical depth in mm. Y-axes depicts the visuomotor index.

b: Choice classification accuracy for superficial (electrodes 1-8) and deep (9-16) as a function of time for all RTs when aligned to checkerboard cue onset for monkey T(top panel) and monkey O (bottom panel). The classification was performed on a session-by-session basis, and the number of units used for superficial and deep electrodes was equalized by setting the number of units used in the classifier to be the same. We only used sessions where we had greater than 10 units recorded from the U-probes in this classification analysis (19 sessions for T, 12 sessions for O). We used 50 ms bins stepped by 2 ms bins and used a linear classifier.

Supp. Fig. 13 - Single and Multiunits give the same results

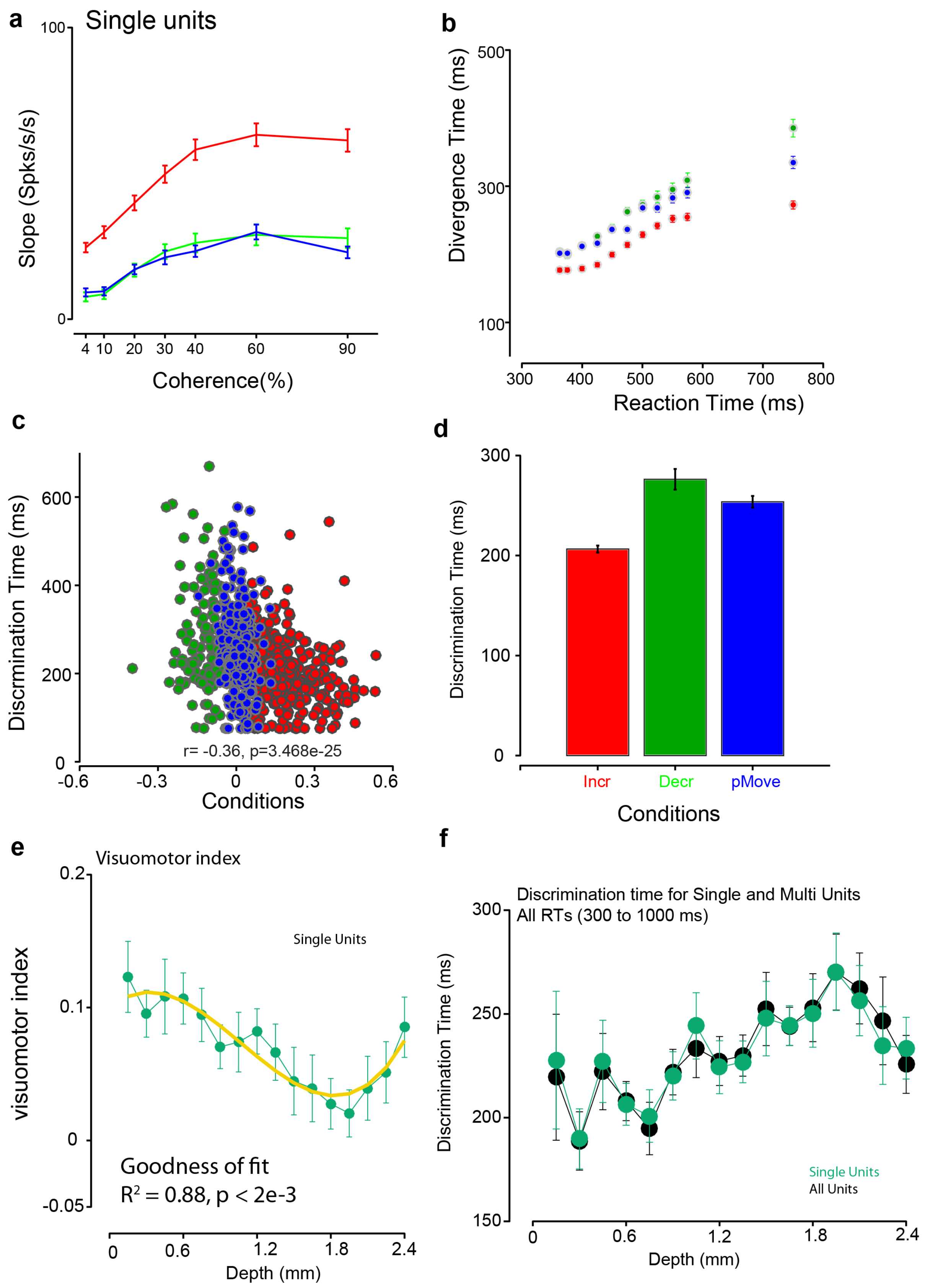

309

Supplementary Figure 13 – Main results are preserved even when analysis is restricted to the single units

a: Slope of the choice selectivity signal (as shown in b) in the 150 – 350 ms epoch (for e.g. demarcated by the shaded grey region in b) after checkerboard cue onset for increased (398 neurons), decreased (124 neurons) and perimovement *neurons* (279 neurons) in PMd for the seven different color coherence levels. Red color denotes increased neurons. Blue and green colors denote the perimovement and decreased neurons. Error bars are SEM estimated over units. We compared the slopes of these curves to a slope estimated through shuffling across color coherences. Please also see Fig. 3c which plots it for both single neurons and multiunits we recorded.

b: The population response of increased neurons (shown in red) begins to signal the eventual choice ~100-150 ms after checkerboard cue onset regardless of RT. Conventions as in Supp. Fig. 4d which shows the same analysis when including both single neurons and multiunits.

c: Scatter plot of the visuomotor index vs. the discrimination time colored by the category of the neuron. There is a significant negative correlation between the index and the time of discrimination. Please see bottom panel of Supp. Fig. 4d for the same analysis that includes both single- and multi-units

d:Average discrimination time for all RTs ranging from 300 to 1000 ms for each broad neuronal category. Please see middle panel of Supp. Fig. 4d for the same analysis that includes both single and multiunits.

e: The visuomotor index estimated only for single neurons by pooling over sessions from both monkeys T and O decreases as a function of depth (68 sessions). X-axes depict depth in mm. Y-axes depict the index. Error bars denote SEM. Figure conventions as in Fig. 4c. Both single neurons and multiunits are shown in the figure. Results are very similar for both single neurons and multiunits.

f: Average discrimination time as a function of cortical depth for the population of PMd neurons. X-axis depicts depth in mm. Y-axis the discrimination time in ms. Errorbars denote SEM estimated over sessions. Both single neurons and multiunits are shown in the figure. Results are very similar for both single neurons and multiunits.

Supplementary Note 1: Testing candidate computational models to describe the behavior of the monkeys

The behavior of the monkeys can be explained by both the DDM and the UGM

To assist in the interpretation of decision-related neural responses in PMd, we examined the behavior of the monkeys using two different behavioral models proposed for decision-making, the drift-diffusion model (DDM)[1, 6] and the urgency gating model (UGM)[7, 8, 9]. The first model, the DDM is a well established model that was initially proposed in landmark psychophysical studies to explain choice and RT[1, 6] especially in tasks where the evidence provided by a sensory stimulus was constant over time (such as our static visual checkerboard). The DDM has since then been advanced as a candidate framework to understand FRs in many brain regions during perceptual decision-making[10, 11, 12, 13, 14, 15, 16]. The second model, the UGM was recently proposed to provide a better description of both behavior and PMd responses in decision-making tasks with dynamically changing stimuli[7, 8, 9, 17]. Subsequent studies have suggested that the UGM can explain behavior even in tasks with constant evidence stimuli[18]. Both models predict lawful but different structures for RT and behavioral accuracy and we examined if the behavior of the monkeys were better described by one or the other model.

We first fit the monkeys' RT distributions and accuracy with a full variant of the DDM. The assumption of the DDM is that choice behavior and RTs arise from a process of deliberation that involves the integration of evidence (e.g. noisy spike trains in sensory areas in response to the red and green squares in the checkerboard cue) to one of two predefined bounds[19, 20, 21]. This DDM incorporates both variability in the drift-rate as well as the non-decision time parameter (Supp. Fig. 1a)[19, 20, 22, 23, 24]. Comparison models that did not incorporate variability in these parameters were substantially poorer fits to the behavior. The goodness of fit statistics for the different variants of the DDM that we fit to the data are shown in Supp. Table 1.

As color coherence decreases, the DDM predicts poorer discrimination accuracy and longer RTs (Supp. Figs. 1b-c)—observations consistent with measured behavior shown in Figs. 1d, e. In Supp. Figs. 1b, c the magenta points denote the DDM fits and the black filled points either show the measured proportion responded red or measured RTs. Magenta line segments are drawn in between the predicted points from the DDM to guide the eye.

Consistent with prior studies in humans and monkeys using similar stimuli[23, 24, 25, 26, 27], the model fits from the DDM, estimated using choice behavior and RT distributions pooled all sessions, were excellent for both monkeys (>100,000 trials per monkey, Supp. Figs. 1b-c, Psychometric curve: R^2=0.99 for T and R^2=0.99 for O; Pooled RT: R^2=0.98 for T; R^2=0.91 for O). The model fits to the mean RT were better for the easier coherences than the harder coherences. The DDM, as previously noted, overestimates the RTs for the hardest coherences[26]. Differences in discrimination behavior and RT of the monkeys were explained by smaller bound height parameters for monkey O (A=0.65) compared to monkey T (A=1.02) suggesting that monkey T integrated evidence for a longer duration to make his decision. The parameters of this best fit model are provided in Supp. Tables 2, 3.

We used the mean estimate of non-decision times from the DDM fit and subtracted it from the measured RT to arrive at a rough estimate of decision time ranges (Supp. Fig. 1d, mean of the non-decision time estimate from the model (t_0): 332 ms for monkey T, 364 ms for monkey O).

These non-decision time estimates are quite high. However, we note that in the variant of the DDM that provides the best fit to the behavior, the non-decision times are also modeled as a uniform distribution, and this introduces a trial-to-trial variability for non-decision times[28]. Incorporating this variability in non-decision times in behavioral models is also consistent with the many neurophysiological studies that find that baseline FRs of neurons before stimulus onset in decision-related structures covary with RT[29, 30, 31]. This variability range was ~140 ms for monkey T and ~120 ms for monkey O. So non-decision times could be in principle as low as ~256 ms for monkey T and ~290 ms for monkey O. These non-decision time estimates are similar to reports of mean RTs of ~245 to ~335 ms from simple delayed-reach tasks where the assumption is that these tasks provide access to these non-decision times uncontaminated by other factors[17, 32].

After subtracting out the mean non-decision time, mean decision time estimates from the DDM fit for the hardest checkerboard cues are ~220 ms for monkey T and ~93 ms for monkey O. For monkey T, decision times for a significant proportion of the trials (~35-40%) for the hardest color coherence lie between 220 ms (mean decision-time) to 510 ms (estimated from 95th percentile of the RT distribution). The corresponding range is ~92-227 ms for monkey O. Overall, these estimates of decision time using the DDM for our monkeys overlap with or exceed decision times observed in monkeys performing static visual stimulus discrimination tasks[32, 33] or an RT auditory discrimination task[20], and overlap with estimated decision times for monkeys performing variable duration random-dot discrimination tasks[19, 34]. Our decision time estimates are lower than some estimates of decision times of some monkeys performing RT random-dot motion discrimination tasks[12]. Lower decision times as estimated by the DDM in our task, especially for monkey O, may be due to factors such as the static nature of the stimulus (in comparison to the time-varying nature of the random-dots stimulus which in principle encourages greater amounts of temporal integration), use of arm movements as a behavioral report, and differences in behavioral training.

The DDM is not the only model which has been proposed to explain the behavior of monkeys performing discrimination tasks. The second candidate computational model recently used to describe RTs and accuracy of participants in discrimination tasks is the Urgency Gating Model (UGM)[7, 17, 29]. In the UGM, sensory evidence is not temporally integrated to a bound. Instead, novel sensory evidence is low pass filtered (a fixed time constant of 100 ms was used here) and then multiplied by an urgency signal that grows with time[35]. A response is initiated when a sample exceeds a decision threshold[7, 17, 29]. Using the framework described in ref. [26], we quantitatively examined whether the RT and accuracy of the monkeys were better fit by the UGM or the DDM.

Supp. Fig. 2a shows the fits from a DDM (left panel), a UGM with a time constant of 100 ms (urgency signal = βt, middle panel, β=1), and another UGM with the same 100 ms time constant but also including an additional intercept term for the urgency signal (urgency signal = $\alpha + \beta$t, right panel, β=1). In this framework, the number of free estimated parameters for the DDM and one of the UGMs is the same (7 different drift rates, a non-decision time and an upper bound and trial-by-trial variability in the drift rates). The other UGM also included an additional parameter because of the intercept used for the urgency signal. A closer analysis of the plots presented in the left panel of Supp. Fig. 2a suggests that the DDM is an excellent fit to the lower quantiles of RT (10, 30, 50th percentiles) but overestimates the 70th and 90th percentiles relative to the data (consistent with the inflated mean RTs for the harder coherences shown in Supp. Fig. 1). Although the UGM without an intercept term did not overestimate the RTs for the highest quantiles it demonstrated inflated mean RT s for the error trials and suggested a shape for the RT distribution inconsistent with the behavior of the monkeys. The best model fit of the three models was achieved by the UGM with a 100 ms

time constant and an urgency signal with an intercept term (right panel). The addition of an intercept term to the urgency signal provided it with the flexibility to generate early decisions and thus better model RT distributions. This model provides a shape for the RT distribution which is more consistent with the behavior of the monkeys albeit with one additional parameter. Crucially, the UGM does not over predict the RTs for the highest quantiles and begins to show shapes that are consistent with the shapes for the real data.

These analyses suggest that incorporation of an urgency signal might be needed to explain the behavior of monkeys performing this checkerboard discrimination task [7, 9, 36, 37] and that the UGM with both a slope and intercept term for the urgency signal provides a modestly better description of the behavior of the monkeys performing this checkerboard discrimination task [18].

Supplementary Discussion: Drift diffusion vs. Urgency Gating Model of Decision-making

Our results from the model fitting analysis suggested that the behavior of the monkeys are consistent with some of the predictions from the DDM[23, 24] as well as some predictions of the UGM[8, 17, 18, 38]. However, the DDM consistently predicted longer RTs than what was observed in the behavior — a problem recognized previously in models attempting to describe the behavior of human subjects performing this task[39]. The UGM with an intercept and slope term was the best fit to the RT data of the monkeys.

This analysis first reiterates the well-known value of examining RT distributions to fit computational models of decision-making rather than just the mean RTs and accuracy[1, 26]. Moving forward, incorporating a collapsing threshold for the DDM, different time courses (exponential, parabolic, etc.) for the urgency signals, and adjustments of time constants used in the UGM are elaborations that will likely improve the fits to the RTs and accuracy of the monkeys in this task[25, 36, 37, 39, 40, 41, 42]. Such analyses might help pin down the degree to which monkeys integrate or not the sensory evidence in this checkerboard discrimination task. Consistent with this observation, a recent study showed a threefold improvement in fits to the behavior when using a DDM with collapsing bounds to model the behavior of human subjects performing a variant of the checkerboard discrimination task[39]. Rigorous model comparison by elaborating all of these different model variants and tests of a wide range of parameter regimes is needed if modeling methods are used to resolve this debate about mechanisms underlying perceptual decisions[1, 26].

Besides advances in the mathematical modeling of behavior from decision-making tasks, an important complementary approach to distinguish between the DDM and the UGM will come from using dynamically changing stimuli and also incorporating task designs that involve speed accuracy tradeoffs[8, 17, 18, 38]. The DDM and the UGM make different predictions for dynamically changing stimuli, and thus stimulus manipulations provide a powerful tool to dissociate between these models. A recent set of studies provided an important illustration of the power of these dynamically changing stimuli[17, 29, 35] by adopting a novel "tokens" task where the sensory evidence dynamically changed over time[17]. The behavior of the monkeys and humans in this task were consistent with the UGM and not the DDM[17, 29, 35]. Paralleling this behavioral finding, neural responses in PMd do not appear to integrate sensory samples. Instead, activity in PMd quickly tracks sensory information and combines it with a growing urgency signal[17], and this is consistent with the predictions of UGM. One purported limitation of the "tokens" task design is that the stimulus by itself provides the integral of the sensory evidence[38]. This is in comparison to the random-dots stimuli where only the

momentary evidence is available and not the integral of the sensory evidence. However, recent efforts in humans have addressed this issue by using a variant of the random dot motion discrimination task and suggesting that the UGM might apply in other stimulus contexts as well[18].

Finally, understanding which model describes the neurophysiological responses in PMd and other structures may also emerge from an explicit examination of the recurrent nonlinear dynamics either at the single-neuron single-trial level[43] or at the population level[44, 45]. In addition, examining the same neurons in monkeys trained to perform two different tasks, one with constant evidence (e.g., the static checkerboard, the random dot motion stimuli) and the other with dynamically changing evidence (e.g. the tokens stimuli, or random dot stimuli with shaped stimulus statistics for each set of frames) might provide a way to identify whether there is a universal decision-making mechanism adopted regardless of task context and stimulus[46].

Supplementary Note 2: Discrimination time differences are preserved on a neuron-by-neuron basis

We also performed the discrimination time analysis on a *neuron-by-neuron* basis and observed the same result (Supp. Figs. 4d-f, for the comparisons between increased vs. decreased as well as increased vs. perimovement, all p values for permutation tests for each RT bin over 10000 shuffles were < .009). The average discrimination time for the increased units over all RTs (300-1000 ms) was significantly faster for the increased compared to both the decreased and perimovement units (Wilcoxon ranksum comparing median discrimination time for Increased vs. Decreased, Pooled: p=2.28e-09, Monkey T: 8.97e-05; Monkey O: 5.51e-07; Increased vs. Perimovement: Wilcoxon ranksum, Pooled p=3.457e-14; Monkey T: 4.45e-11, Monkey O: 1.68e-04). Finally, the discrimination time estimated over all RTs (300-1000 ms) was negatively correlated with the visuomotor index (Spearmans's rank correlation pooled r=-0.32, p=2.67e-24, Monkey T: r=-0.36, p=9.188e-18, Monkey O: r=-0.36, p =3.073e-14, bottom panels of Supp. Figs. 4d-f).

Supplementary Note 3: Choice selectivity is distributed in the PMd neural population

We also examined the relationship between visuomotor index and choice selectivity to understand if choice selectivity is distributed or clustered in this PMd neural population. The left panel of Supp. Fig. 4h shows the correlation between choice probability and the visuomotor index when aligned to movement onset. The right panel of Supp. Fig. 4h shows a scatter plot of the visuomotor index vs. choice probability for time points 400, 300 and 200 ms before movement onset. At 300 ms before movement onset, there is a strong covariation between the visuomotor index and choice probability. For all three time points, choice probability is largely consistent with a continuum and less with distinct clusters of high and low choice selectivity.

Supplementary Note 4: Additional metrics for characterizing a visuomotor continuum are consistent with temporal heterogeneity in decision-related PMd responses

We first used the method developed in Ref. [4] to characterize a visuomotor continuum (a schematic is provided in Supp. Fig. 5a). In this approach, for every neuron, a parameter λ that denotes normalized latency (defined as $\frac{\mu_{NL}}{\mu_{RT}}$, where μ_{NL} is mean neuronal latency, μ_{RT} is mean reaction time) and a parameter β that describes the dependence of FRs on RT is estimated. These parameters are estimated using a technique that does not involve solving the difficult problem of estimating single-trial latencies from Poisson spike trains. Simulations of a hypothetical pure visual neuron (a modest λ, β=0) and a hypothetical pure movement neuron (higher λ, β=1) are shown in Supp. Figs. 5b and 5c respectively. Reassuringly, the estimated parameters from applying the algorithm to the spike trains from the hypothetical neurons are close to the generative parameters we used.

Our prediction was that increased neurons should have the lowest λ, β values and the perimovement units would have the largest λ, β values. Our results were largely consistent with this prediction (Supp. Fig. 5d-f). Both λ and β (averaged over both choice directions for each unit) were negatively correlated with the visuomotor index (Spearman's correlation coefficient; pooled: r=-0.26, p=2.99e-17; β: r=-0.30, p=5.59e-22, Monkey T: λ: r=-0.37, p=3.42e-19; β: r=-0.36, p=1.25e-18, Monkey O: λ: r=-0.25, p=7.09e-08; β: r=-0.30, p=5.14e-11). As expected and in part by definition, these values of λ and β were also positively correlated with the discrimination time of the neurons (λ, r=0.21, p=1.44e-11; β: r=0.19, p=2.63e-10)—neurons with lower values of λ (or β) signaled the choice earlier than neurons with higher values of λ (or β). Together these results using the method from[4] are consistent with our findings using the visuomotor index.

Our second approach was to use a novel analysis method developed in ref. [5] to relate neuronal responses to RT. This method uses an iterative method to identify for each trial, an offset that would best align that trial with the average over all the other trials (Supp. Fig. 6a-c). A correlation coefficient is then estimated between these offsets and the RTs (Supp. Figs. 6d, e). In our case, the units that show sustained patterns of FR (either increases or decreases) when aligned to movement onset should show a significant correlation (Supp. Fig. 6d for example). The perimovement units, in contrast, should need minimal shifts as a function of RT (Supp. Fig. 6e). We, therefore, performed this analysis and estimated for each unit the set of offsets needed to maximally align the responses and correlated this trial-by-trial offset to the RT on different trials. Note that this method, unlike our visuomotor index, will estimate significant *positive* correlations for units that decrease their FRs as well as units that increase their FRs. To estimate a sign for this correlation, we added an additional "directionality" term by measuring whether the difference in FR in the 100 ms epoch before movement onset was higher or lower than the FR in the -800 to -700 ms epoch before movement onset. We multiplied the correlation estimated from the method and this directionality term. This sign corrected correlation (averaged over both choice directions for each unit) and the visuomotor index we proposed are closely related to one another (Supp. Fig. 6f, Spearman's r = 0.37, p = 1.36e-33).

The third approach we used to characterize this neural population was to examine the slope of the regression line between the discrimination latency and the RT curve measured on a neuron-

by-neuron basis when aligned to checkerboard cue onset and to movement onset. This analysis is based on the method proposed in refs.[47, 48]. The assumption of this analysis is that a neuron in the "visual" side of the continuum, when aligned to checkerboard cue onset, would show only modest changes in the discrimination time as a function of RT (red line in upper panel of Supp. Fig. 7a). In contrast, the response of a perimovement neuron would closely follow the RT this would lead to a systematic increase in discrimination time with RT (blue line in the upper panel of Supp. Fig. 7a). The slope of these curves aligned to checkerboard cue onset (m_{cue}) would provide an estimate of the position of this neuron along this visuomotor continuum. Similarly, when aligned to movement onset, the opposite pattern should be observed. A hypothetical neuron in the visual portion of the continuum should show earlier discrimination time relative to movement onset for longest RTs (red line in the lower panel of Supp. Fig. 7a). The hypothetical movement neuron should show the same discrimination time as a function of RT (blue line in the lower panel of Supp. Fig. 7a). The slope of these curves as a function of RT when aligned to movement onset also provides yet another description of the position of neurons along the continuum. For a canonical visual neuron, we would have m_{cue}~0 and m_{move}~-1. A canonical movement neuron should have m_{cue}~1, m_{move}~0. Intermediate values shown in the figure in gray lines are meant to depict different neurons in the visuomotor continuum. Supp. Fig. 7b shows the distribution of the m_{cue} and m_{move} slopes for the population of units investigated here. Supp. Figs. 7c, 7d show the median slopes for m_{cue} and m_{move} for the population of increased, decreased, and perimovement units. The increased units are the ones with the lowest m_{cue} and negative m_{move}, whereas both the perimovement and decreased neurons are more in the motor end of the continuum with larger m_{cue} and positive m_{move} values (ranksum test, m_{cue}: Increased vs. Decreased: $p = 3e-4$, Increased vs. Perimovement: $p =6.69e-4$; m_{move}: Increased vs. Decreased: $p=3.16e-10$, Increased vs. Perimovement: $p=6.31e-06$, shuffle tests, $p < 0.005$ for all Increased vs. Decreased and Increased vs. Perimovement comparisons). These pairwise comparisons were also consistent with significant negative correlations between the visuomotor index and the slopes suggesting that the increased neurons had lower m_{cue} slopes and more negative m_{move} slopes (m_{cue} vs. visuomotor index, Pooled: $r=-0.15$, $p = 3.33e-6$; Monkey T: $r = -0.17$,$p=8.55e-5$; $r=-0.15$, $p=0.003$; m_{move} vs. visuomotor index , Pooled:, $r = -0.22$, $p = 2.87e-12$; Monkey T: $r = -0.14$, $p = 5.39e-4$; Monkey O: $r=-0.25$, $p=6.05e-8$).

Thus, all of these different analyses that characterize different aspects of the responses bolster the view that a continuum of responses is involved in the decision-formation process in PMd.

Principal component analysis of neural signals suggests that increased units demonstrate stronger covariation with the decision formation process

Our analysis thus far has heavily relied on analysis on a neuron-by-neuron basis to separate out the patterns of FRs in PMd during this decision-making task. However, all of the indices could be clumping together patterns of FRs that were different and thus underestimating the diversity of neural responses during this task. A recent, alternative approach is to use principal component analysis (PCA) to understand the diversity of neural responses and to describe the population level structure in a brain region[46, 49, 50].

Supp. Figs. 8a, b show the first two components extracted by PCA on the population FRs. The first PC involves a general increase in FR after checkerboard cue onset with a modest amount of separation by choice and stimulus coherence. The second principal component has a stronger separation by choice as well as coherence. A plot of the variance vs. the number of dimensions

suggests that a majority of the variance in these PMd FRs is well captured by the first few principal components. The distance in FR between left vs. right reaches for the top 5 PCs that capture ~70% of the variance shows an ordered structure as a function of stimulus coherence (Supp. Fig. 8c).

The loadings on the first two principal components shown in Supp. Fig. 8d provide additional credence to our claim that there are broad unit populations in PMd with different roles in the decision-formation process. If there was a random ensemble of units that we had separated artificially by our index and the other metrics we used above, a plot of the loadings on the first two PCs would be uniformly distributed in this two dimensional space (in that gray shaded circle shown in Supp. Fig. 8d). Instead, we find a non-uniform distribution for the loadings. Both a chi-square test and the more sophisticated PAIRS test[51] developed in a recent study rejected the null hypothesis that the loadings are uniformly distributed on the circle (null hypothesis is that PAIRS index=0, PAIRS index=0.31, p~0, bootstrap test, χ^2 =306.98, p=1.77e-51). We found that the median for the *absolute* value of the loading on PC2 (|PC2|), a component that strongly covaries with choice is larger for the increased compared to the decreased and perimovement units (Ranksum, Increased vs. Decreased, p =0.0013, Increased vs. Perimovement: p=2.38e-13, correlation between absolute loading on PC2 and the visuomotor index: r=0.26, p =1.51e-16). The principal components analysis argues that neurons with increases in FR (projection on PC1) are also the ones that are more likely to have a stronger covariation with choice and coherence (projection on PC2) — a result consistent with the findings from the simple visuomotor index in Fig. 3 (in the main text).

Performing the principal component analysis also provided us with an opportunity to quantify the amount of variance the simple visuomotor index can capture in the data. We found that the visuomotor index was tightly correlated to the loading on the first principal component, the vector that by definition explains the maximal variance. This correlation between the visuomotor index and the first principal component was very large (Supp. Fig. 8e, r=0.83, p~0, spearman's correlation). This first principal component explained ~ 50% of the variance of the FRs and thus our simple index obtained by a regression analysis can account for at least close to half of the variance of the FRs in PMd. Thus, when designing this index, our visual inspection was identifying a property of the FR profile that explained a dominant portion of the FR variance.

Clustering analysis suggests that increased units covary earlier with the decision-formation process

We also adopted a clustering technique as an alternative method to partition with minimal supervision, the patterns of FRs observed in PMd during the decision-making process [52]. Our prediction was that if the increased, decreased and perimovement neurons were meaningful broad populations in PMd, then this should again emerge in the clustering analysis.

Supp. Fig. 9a shows 5 clusters identified through a k-means clustering algorithm. Each panel shows the average FRs of the neurons within a cluster organized by RT, aligned to checkerboard cue onset and separated by the preferred and nonpreferred direction reaches. The size of the cluster and the average visuomotor index and average λ, β values are also provided for each panel. As Supp. Fig. 9a shows, the mean FR for the different clusters is in good agreement with what is predicted by our simple visuomotor index as well as the metrics proposed in ref. [4]. As predicted, the unsupervised clustering technique identifies the decreased units as a cluster (with a negative visuomotor index). It also identifies a "perimovement" like cluster with a visuomotor index that hovers around zero. Finally, the remaining three clusters it identifies appear to involve *increases* in FR (and have positive

visuomotor indices). Supp. Fig. 9b shows the average discrimination time as a function of RT for the different clusters. These latencies are obtained from the neuron-by-neuron estimates of the discrimination time previously plotted in Supp. Fig 4. Supp. Fig. 9c shows the average discrimination time for these neurons across all RTs. Clusters 3 – 5, that are the most consistent with the increased units again show the earliest selectivity for choice.

The question with any clustering analysis is whether the chosen number of clusters is meaningful. To identify the number of clusters, we used the gap statistic method[53]. The gap statistic quantifies the change in within-cluster dispersion with that expected under an appropriate reference null distribution[53]. The number of clusters is chosen to be the minimal value of k that involves the largest separation between the dispersion computed for the true distribution and the reference distribution. With very small numbers of clusters, the difference between the within group dispersion and the reference distribution is low and thus the gap statistic is low. At some point, this difference increases and the first such value is a good indication of a reasonable clustering of the data[53]. A plot of the gap statistic as a function of the number of clusters is shown in Supp. Fig. 9D. As expected for 1 cluster this gap statistic is small. As the number of clusters increases the within group variance begins to decrease, and after ~4-5 clusters the gain in explanatory power by adding a new cluster is modest. If there were really say 100 distinct FR patterns in our population of ~1000 units recorded in PMd during this decision-making task, this gap statistic plot would have no such elbow at ~4-5 clusters. It would smoothly rise until $K = 100$ and then level out or fall off. This clustering analysis again suggests that neurons are consistent with a visuomotor continuum from decreased to increased responses.

There is a modest increase again in the gap statistic for higher numbers of clusters (e.g. $K = 10\text{-}12$). This gap statistic is within 2 standard errors of the gap statistic for $K=5$ suggesting only a modest improvement. We also examined the results from a clustering analysis with 12 clusters and again observe a strong negative correlation between the visuomotor index and the average discrimination time estimated for each of these clusters (Spearman's $r=-0.88$, $p=1.92e\text{-}4$).

Supplementary Note 5: Controls for color of target chosen and hand/eye movement profiles

One potential concern with using selective averaging to show that the FRs of increased units are consistent with a decision variable is that it is circular. Circularity could arise if we only selected units with strong choice selectivity and then performed a statistical test to contend that the selected units are on average strongly choice selective. However, we designed our index to identify units as increased or decreased by only testing if FRs before movement onset increased or decreased as a function of RT. The index did not utilize any information about choice selectivity to separate out units. In support of this assertion, the presence or the absence of covariation with RT before movement onset was the key identifying factor for both increased and decreased units. However, only the increased units had properties consistent with a candidate decision variable.

We also used a regression analysis to examine if there were any relationships between the increased unit FRs and the color of the target chosen by the monkey while controlling for other factors. We built a single regression model in which we included choice, color coherence, and RT as predictors. In the model, we also included the color of the target chosen as a factor as well as other nuisance covariates such as eventual arm movement speed, instantaneous hand position, and eye position on single trials. Choice, RT, and color coherence were again robust and reliable predictors of FRs in increased units (we assessed significance by comparing if confidence intervals of model predictors overlapped with zero). The effects of choice, RT, and coherence were good predictors of FRs in both monkeys. Choice and RT had similar explanatory power for FRs of increased neurons from both monkeys (Supp. Fig. 10c, e). Effects of coherence were stronger in Monkey T than Monkey O.

In contrast, the number of units that significantly covaried with the color of the target chosen was largely at chance levels (~5%) in the epoch before movement onset (Supp. Fig. 10a, c, e). Thus, FRs of increased neurons in PMd do not appear to covary with the color of the target that was chosen—a result consistent with previous observations[32, 54]. Again, this effect was observed in both monkeys (Supp. Fig. 10c, e).

The regression analyses also allowed us to exclude explanations, which propose that response modulations observed in Fig. 3, are due to differences in the kinematics of the eventual arm movement (e.g. speed). For the increased units, the regression analysis showed that nuisance covariates can explain some proportion of FR variance (Supp. Fig. 10b, d, f). However, these nuisance covariates are insufficient to entirely explain the FR variance observed in increased units. Even when these nuisance covariates are included in the regression, choice, color coherence, and RT are still significant predictors of the FRs of increased units (compare Supp. Figs. 10a and 10b). These patterns were again observed in both monkeys. These nuisance covariates explained a more significant fraction of the FR variance in monkey T (Supp. Fig. 10d) than in monkey O (Supp. Fig. 10f).

Supplementary Note 6: Conclusions do not change when examining just isolated single neurons

We also confirmed that our results were not influenced by the inclusion of the multi-units in the database. We examined these effects in four different key analyses that we report in the main results. We focused on the results describing the dependence of slopes on coherence, discrimination time, laminar distribution of the index and discrimination time as a function of cortical depth. None of the conclusions change when only considering the isolated single neurons (Supp. Fig. 13a-f).

First, the dependence on coherence was stronger for the increased compared to the decreased and perimovement neurons (Supp. Fig. 13a, Increased: 45.29 (3.59), Decreased: 25.38 (3.33), Perimovement: 19.09 (1.99), Increased vs. Decreased: Wilcoxon ranksum p = 5.62e-03, increased vs. perimovement: Wilcoxon ranksum p= 2.69e-07, decreased vs. perimovement: Wilcoxon ranksum p = 0.2, correlation between visuomotor index and slope: Spearman's r = 0.33, p < 4.77e-22). Second, increased neurons in PMd signaled the choice earlier than the decreased neurons and perimovement neurons (Supp. Figs. 13b-d, correlation between visuomotor index and discrimination time for all RTs from 300 ms to 1s: Spearman's r=0.36, p < 3.47e-25; Increased vs. Decreased: ranksum p=1.23e-10, increased vs. perimove: p < 8.91e-12). Third, the visuomotor index again showed the same dependence as a function of cortical depth for both single neurons and multi-units (Supp. Fig. 13e, goodness of fit for single neurons: R^2 = 0.88, p < 2e-3, 1000 shuffles). Finally, the discrimination time also increases as a function of cortical depth for only the single neurons (Supp. Fig. 13f, r=0.69, p < 0.0042). Together, the results for single neurons are largely consistent with the results reported including both single neurons and multiunits in PMd.

Supplementary Discussion:

The majority of the electrophysiological data reported in this manuscript were single neurons (~80%) recorded in PMd during the decision-making task. However, we also included a substantial fraction of multiunits in the data (~20%). The multiunits certainly provided us with additional power for the analyses presented here, but it may have also lead to spurious misclassification of units in the continuum. First, in the worst case, there is the possibility of combining the FRs of an increased neuron with a decreased neuron and a perfect cancelling in the decision-formation period could result in a spurious perimovement unit. Fortunately, as our laminar recordings show, the increased, decreased and perimovement like FRs appear to be roughly segregated as a function of cortical depth, so this type of spurious mixing will be minimized due to this topographic organization. Second, because a multiunit contains additional spikes, there is a slightly greater chance that a decreased neuron will be misclassified as a perimovement or an increased unit and this will increase the preponderance of increased units in our dataset. Third, there is also the possibility that some finer grained temporal patterns are smeared because of combining multiple neurons into a unit. Finally, inclusion of the multiunits could have resulted in smoother visuomotor continuum than what is actually present in PMd. Future studies that use a laminar electrode with a tetrode configuration that will improve isolation or the next generation of silicon electrodes that provide high-density recordings in combination with automated sorting methods may further illuminate the microcircuit in PMd and other structures involved in perceptual decision-making[55, 56]. These techniques may allow us to derive so-called "electroanatomical" maps of brain regions[55].

Supplementary Note 7: Recordings in caudal locations of the recording chamber do not show the same laminar trends observed in PMd

We also compared these trends observed in PMd to those recorded in one of the monkeys (monkey T) in more caudal locations of our recording chamber (putative M1, 12 sessions, 95 units). As Supp. Fig. 12a shows, the visuomotor index did not show the same decrease as a function of cortical depth as observed in PMd and if anything appears to increase as a function of cortical depth ($r=0.42$, $p = 0.1$). Thus, at least in one monkey and for a modest population of neurons, we did not observe the same preponderance of increased units in the superficial compared to the deeper layers for the caudal regions of motor cortex.

Supplementary Note 8: Causal experiments are needed to better understand the role of PMd

We have shown that FRs of some PMd neurons demonstrate a very lawful organization to the various aspects of the somatomotor decision-formation process. Our results are similar to the studies that have documented decision-variables in the oculomotor system especially in LIP of monkeys performing random-dot discrimination tasks[12, 19, 34, 36, 57, 58]. What is currently unresolved is the degree of causal involvement of LIP in decision-making [59]. Microstimulation of LIP influences RT and discrimination performance in a visual discrimination task and thus supports a causal role for LIP in decision-making[60]. In contrast, the inactivation of LIP has minimal or no impact on visual-discrimination behavior and these experiments cast doubt on a causal role for LIP in decision-making[61]. This pattern of results in monkeys is also consistent with inactivation experiments in rodents performing auditory accumulation of evidence tasks[62]. FRs of neurons in the posterior parietal cortex (PPC) of rats performing an auditory evidence accumulation task show all the properties of a candidate decision-variable[13]. However, inactivation of the PPC leads to minimal impact on the auditory discrimination behavior[62]. In contrast, the frontal cortex of these same rats show organized FR patterns that covary with various aspects of the decision-formation process and inactivation of these regions does impact discrimination behavior[62]. These effects of inactivation also depend on the stimulus modality used. Inactivation of the posterior parietal cortex can influence behavior in tasks that involve actions based on visual but not auditory stimuli[51, 63].

These discrepancies between correlative physiological experiments and causal inactivation studies in both monkeys and rodents suggest that our and other demonstrations of decision-related activity in PMd cannot be used to identify whether the decision computation itself emerges in PMd[17, 32]. Additional causal experiments that involve inactivation of PMd during a decision-making task are necessary to resolve this question. Inactivation of PMd does impact behavior in simple conditional visuomotor association tasks (e.g. red square means pull a handle, green squares mean push the handle)[64] and in internally generated sequential movement tasks[65]. However, this inactivation study provides no evidence for whether the deliberation on the visual stimulus itself occurs in the microcircuit in PMd. It may be that in contexts that involve ambiguous sensory stimuli (like the decision-making task used here), deliberation occurs in prefrontal and parietal regions and PMd by virtue of its anatomical connections to these areas provides a reflection and thus a neural correlate of these signals[19, 66, 67, 68, 69, 70]. Inactivation or disruption of PMd during the task presented here and tasks using stimuli such as the classical random dot motion patterns or the tokens task will be needed to resolve this question[61, 64, 65, 71, 72].

Further insights into the role of PMd and other prefrontal and parietal brain areas might emerge from inactivation experiments based on the theoretical framework that proposes the existence of a hierarchy of action and cognitive control instantiated along a rostrocaudal gradient in the frontal cortex[73, 74]. In this theoretical framework, neurons in more-anterior regions of the frontal lobe process abstract action goals and those in more caudal regions (e.g. PMd) are more involved in tasks that involve concrete actions based on sensory input (similar to the simple conditional visuomotor association task). Some of the best evidence for this proposal comes from studies that examined the behavior of human patients with lesions in different brain regions along the rostrocaudal gradient using carefully designed tasks that incorporate increasing degrees of abstraction and cognitive control[75, 76]. The findings from these studies is that a PMd lesion impacts tasks that involve immediate concrete actions in response to sensory input as well as tasks that involve higher degrees of cognitive control. In contrast, lesions to regions in the more rostral portion of this gradient (more

in the frontal cortex) only impacted the behavior in tasks that involve greater degrees of abstraction[75, 76]. This asymmetric effect of the lesions suggests an organization for cognitive control where the more caudal regions are necessary nodes for ascending rostral areas and/or for descending processing from the more rostral frontal regions.

Supp. Table 1: Model fit of the DDM to the RT distributions and response accuracy using the fast-dm toolbox

Model	Monkey T (χ^2)	Monkey O (χ^2)
No variability in drift rate (sv) or non-decision-times (st0)	12778	15339
No variability in non- decision times but variable drift rates (sv)	12673	15339
No variability in drift rates but with variability in non decision-times (st0)	3575.1	2235.4
**DDM with trial-by-trial variability for drift-rates and non-decision time (best model fit)**	2316.9	2064.4

Supp. Table 2: Model parameters for the best fitting DDM (measured using the chi-squared values from Supp. Table 1) with trial-by-trial variability for drift-rates and non-decision time are shown below. No variability in start point was assumed. The drift rates for the 14 different signed coherences are shown in Supp. Table 2, and the remaining parameters are shown in Supp. Table 3.

**Signed Coherence**	**-90.22**	**-60**	**-40.44**	**-30.67**	**-20.00**	**-10.22**	**-4**	**4**	**10.22**	**20.00**	**30.67**	**40.44**	**60.00**	**90.22**
**Drift rates (v) for T**	-5.40	-5.48	-4.77	-4.30	-3.21	-1.88	-0.88	0.50	1.58	2.91	4.01	4.72	5.44	5.33
**Drift rates (v) for O**	-4.83	-5.28	-4.66	-4.28	-3.40	-2.24	-1.27	0.22	1.29	2.85	4.22	4.98	5.60	5.04

Supp. Table 3: Parameter names and Interpretations are taken from (http://www.psychologie.uni-heidelberg.de/ae/meth/fast-dm/). For comparison, the non-decision times of the monkeys from [17] are (mean ± SD: 291±40 ms, 335±93 ms).

**Other Parameters**	**Interpretation**	**Monkey T**	**Monkey O**
Threshold Separation (*a*)	The amount of information that is considered for a decision. Large values indicate more conservativeness for making a decision.	1.025	0.65
Relative Starting Point (*zr*)	Indicator of an a priori bias in decision making. When the relative starting point zr deviates from 0.5, the amount of information necessary for a decision differs between response alternatives.	0.529	0.5
Non-decision time (*t0*)	This is the average duration of all non-decisional processes (encoding and response execution).	332 ms	363.5 ms
Inter-Trial-Variability of Non-Decisional Components (*st0*)	This parameter describes the range of a uniform distribution with mean *t0* describing the distribution of actual *t0* values across trials.	142.5 ms<br><br>Leads to non-decision times ranging from ~260 to 403 ms)	142.3<br><br>Leads to non-decision times ranging from (~282 to 434 ms)
Inter-Trial-Variability of Drift (*sv*)	This quantity provides the standard deviation of a normal distribution with mean *v* describing the distribution of actual drift rates from specific trials.	1.14	1.34
Differences in Speed of Response Execution (*d*)	Positive values indicate that response execution is faster for	1.45 ms	1.6 ms

	responses linked to the upper threshold (coded as 1 in *fast-dm*) than for responses linked to the lower threshold.		

Supp. Table 4: Parameters for the UGM and the DDM used in Supp. Fig. 2. For further details of the behavior models please refer to ref. [25]. No variability was assumed in the non-decision time for these variants.

Parameters	Parameters
**DDM**	• **Drift rates for 7 coherences (7),** v_{1-7}<br>• **Upper bound for the model (aU),**<br>• Lower Bound for the model (constant, 0)<br>• z – start point (aU/2)<br>• **Non decision time (T_{er})**<br>• **Trial by trial variability for drift rates (η)**<br>• **Total: 10 estimated parameters, 2 non estimated parameters,**
**UGM without an intercept term**	• **Drift rates for 7 coherences (7),** v_{1-7}<br>• **Upper bound for the model (aU),**<br>• Lower bound for the model (-aU)<br>• z – start point (0)<br>• **Non decision time (T_{er})**<br>• **Trial by trial variability (η)**<br>• **Time constant: 100 ms**<br>• **Urgency signal U(t) = bt, b = 1 as in [35]**<br>• **Total: 10 estimated parameters, 3 non estimated parameters**.
**UGM with an intercept term**	• Same as the UGM above and also an estimated intercept term for the urgency model.<br>• **Urgency signal U(t) = a + bt, b = 1 as in [35].**<br>• **Total of 11 estimated parameters and 3 non estimated parameters**

References

1. Ratcliff R, McKoon G. The diffusion decision model: theory and data for two-choice decision tasks. *Neural Comput* **20**, 873-922 (2008).
2. Ratcliff R, Smith PL, Brown SD, McKoon G. Diffusion Decision Model: Current Issues and History. *Trends Cogn Sci* **20**, 260-281 (2016).
3. Heathcote A, Brown S, Cousineau D. QMPE: estimating Lognormal, Wald, and Weibull RT distributions with a parameter-dependent lower bound. *Behav Res Methods Instrum Comput* **36**, 277-290 (2004).
4. DiCarlo JJ, Maunsell JH. Using neuronal latency to determine sensory-motor processing pathways in reaction time tasks. *J Neurophysiol* **93**, 2974-2986 (2005).
5. Erlich JC, Bialek M, Brody CD. A cortical substrate for memory-guided orienting in the rat. *Neuron* **72**, 330-343 (2011).
6. Ratcliff R. Theory of Memory Retrieval. *Psychological Review* **85**, 59-108 (1978).
7. Thura D, Beauregard-Racine J, Fradet CW, Cisek P. Decision making by urgency gating: theory and experimental support. *J Neurophysiol* **108**, 2912-2930 (2012).
8. Carland MA, Thura D, Cisek P. The urgency-gating model can explain the effects of early evidence. *Psychon Bull Rev* **22**, 1830-1838 (2015).
9. Thura D, Cisek P. On the difference between evidence accumulator models and the urgency gating model. *J Neurophysiol* **115**, 622-623 (2016).
10. Ding L, Gold JI. Neural correlates of perceptual decision making before, during, and after decision commitment in monkey frontal eye field. *Cereb Cortex* **22**, 1052-1067 (2012).
11. Shadlen MN, Newsome WT. Neural Basis of a Perceptual Decision in the Parietal Cortex (Area LIP) of the Rhesus Monkey. *J Neurophysiol* **86**, 1916-1936 (2001).
12. Roitman JD, Shadlen MN. Response of neurons in the lateral intraparietal area during a combined visual discrimination reaction time task. *J Neurosci* **22**, 9475-9489 (2002).
13. Hanks TD, Kopec CD, Brunton BW, Duan CA, Erlich JC, Brody CD. Distinct relationships of parietal and prefrontal cortices to evidence accumulation. *Nature* **520**, 220-223 (2015).
14. Brody CD, Hanks TD. Neural underpinnings of the evidence accumulator. *Curr Opin Neurobiol*, (2016).
15. Murakami M, Vicente MI, Costa GM, Mainen ZF. Neural antecedents of self-initiated actions in secondary motor cortex. *Nat Neurosci* **17**, 1574-1582 (2014).
16. Purcell BA, Heitz RP, Cohen JY, Schall JD, Logan GD, Palmeri TJ. Neurally constrained modeling of perceptual decision making. *Psychol Rev* **117**, 1113-1143 (2010).
17. Thura D, Cisek P. Deliberation and commitment in the premotor and primary motor cortex during dynamic decision making. *Neuron* **81**, 1401-1416 (2014).
18. Carland MA, Marcos E, Thura D, Cisek P. Evidence against perfect integration of sensory information during perceptual decision making. *J Neurophysiol* **115**, 915-930 (2016).
19. de Lafuente V, Jazayeri M, Shadlen MN. Representation of accumulating evidence for a decision in two parietal areas. *J Neurosci* **35**, 4306-4318 (2015).
20. Tsunada J, Liu AS, Gold JI, Cohen YE. Causal contribution of primate auditory cortex to auditory perceptual decision-making. *Nat Neurosci* **19**, 135-142 (2016).
21. Smith PL, Ratcliff R. Psychology and neurobiology of simple decisions. *Trends Neurosci* **27**, 161-168 (2004).
22. Voss A, Nagler M, Lerche V. Diffusion models in experimental psychology: a practical introduction. *Exp Psychol* **60**, 385-402 (2013).
23. Ratcliff R, Thapar A, McKoon G. A diffusion model analysis of the effects of aging on brightness discrimination. *Percept Psychophys* **65**, 523-535 (2003).

24. Voss A, Rothermund K, Brandtstädter J. Interpreting ambiguous stimuli: Separating perceptual and judgmental biases. *Journal of Experimental Social Psychology* **44**, 1048-1056 (2008).
25. Hawkins GE, Forstmann BU, Wagenmakers EJ, Ratcliff R, Brown SD. Revisiting the evidence for collapsing boundaries and urgency signals in perceptual decision-making. *J Neurosci* **35**, 2476-2484 (2015).
26. Hawkins GE, Wagenmakers EJ, Ratcliff R, Brown SD. Discriminating evidence accumulation from urgency signals in speeded decision making. *J Neurophysiol* **114**, 40-47 (2015).
27. Boehm U, Hawkins GE, Brown S, van Rijn H, Wagenmakers EJ. Of monkeys and men: Impatience in perceptual decision-making. *Psychon Bull Rev* **23**, 738-749 (2016).
28. Ratcliff R, Tuerlinckx F. Estimating parameters of the diffusion model: approaches to dealing with contaminant reaction times and parameter variability. *Psychon Bull Rev* **9**, 438-481 (2002).
29. Thura D, Cos I, Trung J, Cisek P. Context-dependent urgency influences speed-accuracy trade-offs in decision-making and movement execution. *J Neurosci* **34**, 16442-16454 (2014).
30. Thura D, Cisek P. Modulation of Premotor and Primary Motor Cortical Activity during Volitional Adjustments of Speed-Accuracy Trade-Offs. *J Neurosci* **36**, 938-956 (2016).
31. Snyder LH, Dickinson AR, Calton JL. Preparatory delay activity in the monkey parietal reach region predicts reach reaction times. *J Neurosci* **26**, 10091-10099 (2006).
32. Coallier E, Michelet T, Kalaska JF. Dorsal premotor cortex: neural correlates of reach target decisions based on a color-location matching rule and conflicting sensory evidence. *J Neurophysiol* **113**, 3543-3573 (2015).
33. Ratcliff R, Hasegawa YT, Hasegawa RP, Smith PL, Segraves MA. Dual diffusion model for single-cell recording data from the superior colliculus in a brightness-discrimination task. *J Neurophysiol* **97**, 1756-1774 (2007).
34. Kiani R, Hanks TD, Shadlen MN. Bounded integration in parietal cortex underlies decisions even when viewing duration is dictated by the environment. *J Neurosci* **28**, 3017-3029 (2008).
35. Cisek P, Puskas GA, El-Murr S. Decisions in changing conditions: the urgency-gating model. *J Neurosci* **29**, 11560-11571 (2009).
36. Churchland AK, Kiani R, Shadlen MN. Decision-making with multiple alternatives. *Nat Neurosci* **11**, 693-702 (2008).
37. Drugowitsch J, Moreno-Bote R, Churchland AK, Shadlen MN, Pouget A. The cost of accumulating evidence in perceptual decision making. *J Neurosci* **32**, 3612-3628 (2012).
38. Winkel J, Keuken MC, van Maanen L, Wagenmakers EJ, Forstmann BU. Early evidence affects later decisions: why evidence accumulation is required to explain response time data. *Psychon Bull Rev* **21**, 777-784 (2014).
39. Coallier E, Kalaska JF. Reach target selection in humans using ambiguous decision cues containing variable amounts of conflicting sensory evidence supporting each target choice. *J Neurophysiol* **112**, 2916-2938 (2014).
40. Ditterich J. Evidence for time-variant decision making. *Eur J Neurosci* **24**, 3628-3641 (2006).
41. Hanks TD, Mazurek ME, Kiani R, Hopp E, Shadlen MN. Elapsed decision time affects the weighting of prior probability in a perceptual decision task. *J Neurosci* **31**, 6339-6352 (2011).
42. Murphy PR, Boonstra E, Nieuwenhuis S. Global gain modulation generates time-dependent urgency during perceptual choice in humans. *Nature communications* **7**, 13526 (2016).
43. Latimer KW, Yates JL, Meister ML, Huk AC, Pillow JW. NEURONAL MODELING. Single-trial spike trains in parietal cortex reveal discrete steps during decision-making. *Science* **349**, 184-187 (2015).

44. Yu BM, Cunningham JP, Santhanam G, Ryu SI, Shenoy KV, Sahani M. Gaussian-process factor analysis for low-dimensional single-trial analysis of neural population activity. *J Neurophysiol* **102**, 614-635 (2009).
45. Afshar A, Santhanam G, Yu BM, Ryu SI, Sahani M, Shenoy KV. Single-trial neural correlates of arm movement preparation. *Neuron* **71**, 555-564 (2011).
46. Mante V, Sussillo D, Shenoy KV, Newsome WT. Context-dependent computation by recurrent dynamics in prefrontal cortex. *Nature* **503**, 78-84 (2013).
47. Sato TR, Schall JD. Effects of stimulus-response compatibility on neural selection in frontal eye field. *Neuron* **38**, 637-648 (2003).
48. Song JH, McPeek RM. Roles of narrow- and broad-spiking dorsal premotor area neurons in reach target selection and movement production. *J Neurophysiol* **103**, 2124-2138 (2010).
49. Shenoy KV, Kaufman MT, Sahani M, Churchland MM. A dynamical systems view of motor preparation: implications for neural prosthetic system design. *Prog Brain Res* **192**, 33-58 (2011).
50. Churchland MM*, et al.* Neural population dynamics during reaching. *Nature* **487**, 51-56 (2012).
51. Raposo D, Kaufman MT, Churchland AK. A category-free neural population supports evolving demands during decision-making. *Nat Neurosci* **17**, 1784-1792 (2014).
52. Meister ML, Hennig JA, Huk AC. Signal multiplexing and single-neuron computations in lateral intraparietal area during decision-making. *J Neurosci* **33**, 2254-2267 (2013).
53. Tibshirani R, Walther G, Hastie T. Estimating the number of clusters in a data set via the gap statistic. *Journal of the Royal Statistical Society: Series B (Statistical Methodology)* **63**, 411-423 (2001).
54. Cisek P, Kalaska JF. Neural correlates of reaching decisions in dorsal premotor cortex: specification of multiple direction choices and final selection of action. *Neuron* **45**, 801-814 (2005).
55. Berenyi A*, et al.* Large-scale, high-density (up to 512 channels) recording of local circuits in behaving animals. *J Neurophysiol* **111**, 1132-1149 (2014).
56. Rossant C*, et al.* Spike sorting for large, dense electrode arrays. *Nat Neurosci* **19**, 634-641 (2016).
57. Gold JI, Shadlen MN. The neural basis of decision making. *Annu Rev Neurosci* **30**, 535-574 (2007).
58. Bennur S, Gold JI. Distinct representations of a perceptual decision and the associated oculomotor plan in the monkey lateral intraparietal area. *J Neurosci* **31**, 913-921 (2011).
59. Pisupati S, Chartarifsky L, Churchland AK. Decision Activity in Parietal Cortex - Leader or Follower? *Trends Cogn Sci* **20**, 788-789 (2016).
60. Hanks TD, Ditterich J, Shadlen MN. Microstimulation of macaque area LIP affects decision-making in a motion discrimination task. *Nat Neurosci* **9**, 682-689 (2006).
61. Katz LN, Yates JL, Pillow JW, Huk AC. Dissociated functional significance of decision-related activity in the primate dorsal stream. *Nature* **535**, 285-288 (2016).
62. Erlich JC, Brunton BW, Duan CA, Hanks TD, Brody CD. Distinct effects of prefrontal and parietal cortex inactivations on an accumulation of evidence task in the rat. *eLife* **4**, (2015).
63. Harvey CD, Coen P, Tank DW. Choice-specific sequences in parietal cortex during a virtual-navigation decision task. *Nature* **484**, 62-68 (2012).
64. Kurata K, Hoffman DS. Differential effects of muscimol microinjection into dorsal and ventral aspects of the premotor cortex of monkeys. *J Neurophysiol* **71**, 1151-1164 (1994).

65. Ohbayashi M, Picard N, Strick PL. Inactivation of the Dorsal Premotor Area Disrupts Internally Generated, But Not Visually Guided, Sequential Movements. *J Neurosci* **36**, 1971-1976 (2016).
66. Tanji J, Hoshi E. Role of the lateral prefrontal cortex in executive behavioral control. *Physiological reviews* **88**, 37-57 (2008).
67. Hoshi E. Cortico-basal ganglia networks subserving goal-directed behavior mediated by conditional visuo-goal association. *Frontiers in neural circuits* **7**, 158 (2013).
68. Yamagata T, Nakayama Y, Tanji J, Hoshi E. Distinct information representation and processing for goal-directed behavior in the dorsolateral and ventrolateral prefrontal cortex and the dorsal premotor cortex. *J Neurosci* **32**, 12934-12949 (2012).
69. Swaminathan SK, Freedman DJ. Preferential encoding of visual categories in parietal cortex compared with prefrontal cortex. *Nat Neurosci* **15**, 315-320 (2012).
70. Swaminathan SK, Masse NY, Freedman DJ. A comparison of lateral and medial intraparietal areas during a visual categorization task. *J Neurosci* **33**, 13157-13170 (2013).
71. Halsband U, Passingham R. The role of premotor and parietal cortex in the direction of action. *Brain Res* **240**, 368-372 (1982).
72. Passingham RE. Cues for movement in monkeys (Macaca mulatta) with lesions in premotor cortex. *Behavioral neuroscience* **100**, 695-703 (1986).
73. Badre D, D'Esposito M. Is the rostro-caudal axis of the frontal lobe hierarchical? *Nat Rev Neurosci* **10**, 659-669 (2009).
74. Voytek B*, et al.* Oscillatory dynamics coordinating human frontal networks in support of goal maintenance. *Nat Neurosci* **18**, 1318-1324 (2015).
75. Badre D, Hoffman J, Cooney JW, D'Esposito M. Hierarchical cognitive control deficits following damage to the human frontal lobe. *Nat Neurosci* **12**, 515-522 (2009).
76. Azuar C*, et al.* Testing the model of caudo-rostral organization of cognitive control in the human with frontal lesions. *Neuroimage* **84**, 1053-1060 (2014).
